# Genome-wide analysis of histone modifiers in tomato: gaining an insight into their developmental roles

**DOI:** 10.1186/1471-2164-14-57

**Published:** 2013-01-28

**Authors:** Riccardo Aiese Cigliano, Walter Sanseverino, Gaetana Cremona, Maria R Ercolano, Clara Conicella, Federica M Consiglio

**Affiliations:** 1 CNR, National Research Council of Italy, Institute of Plant Genetics, Research Division Portici, Via Università 133, 80055, Portici, Italy; 2 DISSPAPA, Department of Soil, Plant and Environmental Sciences, University of Naples “Federico II”, Via Università 100, 80055, Portici, Italy

**Keywords:** *Solanum lycopersicum*, Epigenetics, Development

## Abstract

**Background:**

Histone post-translational modifications (HPTMs) including acetylation and methylation have been recognized as playing a crucial role in epigenetic regulation of plant growth and development. Although *Solanum lycopersicum* is a dicot model plant as well as an important crop, systematic analysis and expression profiling of histone modifier genes (*HMs*) in tomato are sketchy.

**Results:**

Based on recently released tomato whole-genome sequences, we identified *in silico* 32 histone acetyltransferases (HATs), 15 histone deacetylases (HDACs), 52 histone methytransferases (HMTs) and 26 histone demethylases (HDMs), and compared them with those detected in Arabidopsis (*Arabidopsis thaliana*), maize (*Zea mays*) and rice (*Oryza sativa*) orthologs. Comprehensive analysis of the protein domain architecture and phylogeny revealed the presence of non-canonical motifs and new domain combinations, thereby suggesting for HATs the existence of a new family in plants. Due to species-specific diversification during evolutionary history tomato has fewer HMs than Arabidopsis. The transcription profiles of *HMs* within tomato organs revealed a broad functional role for some *HMs* and a more specific activity for others, suggesting key *HM* regulators in tomato development. Finally, we explored *S. pennellii* introgression lines (ILs) and integrated the map position of *HMs*, their expression profiles and the phenotype of ILs. We thereby proved that the strategy was useful to identify *HM* candidates involved in carotenoid biosynthesis in tomato fruits.

**Conclusions:**

In this study, we reveal the structure, phylogeny and spatial expression of members belonging to the classical families of HMs in tomato. We provide a framework for gene discovery and functional investigation of *HMs* in other *Solanaceae* species.

## Background

Chromatin is characterized by a dynamic multi-level organization passing through the nucleosomal basic unit, the 30-nm fiber, and higher-order folding up to the chromosome [1]. Nucleosome remodeling, histone post-translational modifications (HPTMs), DNA methylation, and other factors contribute to define different chromatin states which drive transcription and other chromatin-based nuclear processes [2-4]. In particular, HPTMs correlate largely with transcriptional regulation, but they are also involved in DNA replication, histone deposition, and DNA repair and recombination. HPTMs occurring in core histone tails include a variety of covalent modifications including acetylation, methylation, phosphorylation, and ubiquitination [2]. Histone acetylation is a reversible process carried out by two classes of enzymes known as histone acetylases (HATs) and histone deacetylases (HDACs) acting on the ε-amino group of lysine residues in histones. The acetylation targets in the H3 tail are lysine (K) residues 9, 14, 18 and 23, and in H4 lysine (K) residues 5, 8, 12, 16 and 20 [5].

HATs and HDACs are classified into different families that are generally conserved in eukaryotes, including yeast, animals, and plants. Plant HATs are currently categorized into four groups on the basis of homology with other eukaryotic HATs and domain composition: (i) HAG with Acetyltransf_1 domain (PF00583) (AT1) include GCN5-, ELP3-, HAT1-like acetyltransferases; (ii) HAM with a MYST (MOZ-YBF2/SAS3-SAS2-TIP60) domain; (iii) HAC with similarity to p300/CREB-binding protein; (iv) HAF related to the TATA binding protein-associated factor 1 [6]. Specific HATs acetylate H4K5 (HAM members), H4K12 and H3K14 (HAG members). Other acetylation marks, including H3K9, are likely to result from the activities of HAC members with broad specificity [7]. Plant HDACs are grouped into three families: RDP3/HDA1, hereinafter named HDAs, SIR2 and HD2. Two of these families are homologous to HDACs found in yeast and animals while the HD2 family appears to be unique to plants and unrelated to the other families [6]. Of all HPTMs, acetylation has the most potential to unfold chromatin since it neutralizes the basic charge of the lysine [2]. In the histones, HDACs remove acetyl groups added by HATs by resetting the chromatin structure for the transcription. Furthermore, HDACs and HATs can function in protein complexes as transcriptional co-repressors and co-activators [8-10] or associated with chromatin remodelers as modulators of the accessibility of DNA to different machineries.

In Arabidopsis, HDACs and HATs are emerging as crucial players in growth and development processes, including meiotic recombination, embryogenesis, flowering, and senescence as well as in responses to environmental cues [11,12]. While histone acetylation is dynamically regulated by HATs and HDACs, histone methylation is balanced by the activities of histone methylases (HMTs) and histone demethylases (HDMs) [13]. Plant histone methyltransferases are assigned to different protein groups based on sequence similarity with SET domains (SDG) found in *Drosophila* SU(VAR)3-9, Enhancer of Zeste E(z), Tritorax (TRX), and absent, small, or homeotic discs (ASH1). These proteins function in covalent addition of one (me1), two (me2) or three (me3) methyl groups to lysine residues in histone tails H3K4, H3K9, H3K27, H3K36, and H4K20. Arabidopsis SU(VAR)3-9 members have H3K9 methyltransferase activity and play a key function predominantly in heterochromatin formation and gene silencing [14-18]. Enhancer of Zeste E(z) proteins catalyze H3K27 trimethylation and are involved in the repressive control of gene expression [19]. TRX proteins mediate H3K4 methylation and are required for transcriptional gene activation as well as ASH1 proteins that have a dual methyltransferase function for both H3K4 and H3K36 [20]. Histone methylation occurs also at the arginine residues and is catalyzed by protein arginine methyltransferase (PRMTs) [21]. Arabidopsis histone methyltransferases and their importance in relation to plant development have recently been reviewed [22]. Histone methylation has long been regarded as an irreversible mark until the discoveries in mammals of two families of HDMs, KDM1 (histone lysine demethylase 1) also known as lysine-specific demethylase 1 (LSD1) and the JmjC domain (Jumonji C) containing proteins [23]. Arabidopsis homologs of human LSD1 act to reduce the level of H3K4 methylation. They were discovered at the level of floral repressor *FLOWERING LOCUS C (FLC)*, a key component of a regulatory network that controls the timing of the start of flowering [24]. The JmjC proteins are able to remove the methyl group on H3K4, H3K9, H3K27 and H3K36 [25,26]. Unlike KDM1, these proteins could reverse all the states (me1, me2, me3) of lysine methylation [21,27-29]. Furthermore, a member of the JmjC proteins has been shown to demethylate arginine H3R2 and H4R3 in animal cells [30]. However, arginine demethylase activity remains to be determined in plants. Recent studies have revealed a role for Arabidopsis JmjC proteins in several aspects of plant development such as floral transition [26,28,29], gametophyte function [31], and circadian rhythm [32,33].

In spite of the crucial role emerging for epigenetic modifications in plant growth and development, little is known regarding HMs in the important crop *Solanum lycopersicum*. Using the complete sequence of tomato genome as well as transcriptomes at different stages/organs [34] we investigated *HM* genes through a bioinformatic approach. In this study we give a comprehensive overview of the structure, phylogeny and spatial expression of members belonging to the classical families of HMs in tomato. Furthermore, we shed light on the position of *HMs* on the tomato genome. We combined this information and *HM* expression profiles with the phenotype of tomato introgression lines (ILs) in order to identify candidate genes involved in epigenetically regulated processes.

## Results and discussion

In this study, 124 histone modifiers (HMs) were identified in tomato. We systematically classified 32 proteins belonging to HATs, 14 to HDACs, 52 to HMTs, and 26 to HDMs.

### Tomato HATs

#### HAGs

**Figure 1:**
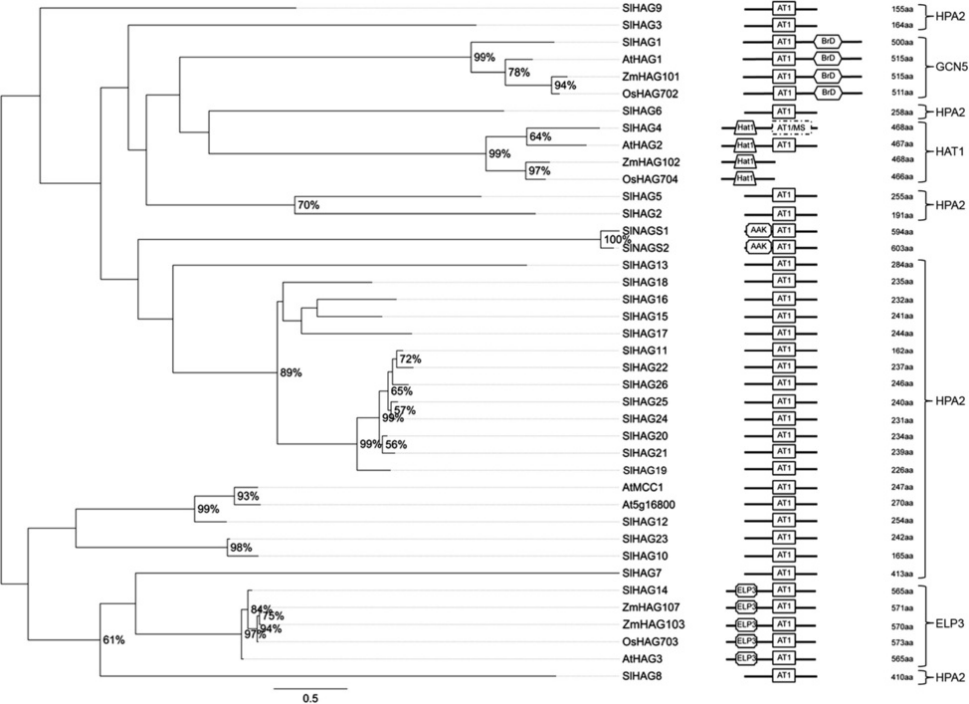
**Phylogenetic tree and domain composition of HAG proteins.** Maximum likelihood phylogenetic tree of HAG predicted proteins from *Arabidopsis thaliana* (At), *Oryza sativa* (Os), *Solanum lycopersicon* (Sl) and *Zea mays* (Zm). Bootstrap values higher than 50% are shown. The tree is drawn to scale, with branch lengths measured in the number of substitutions per site. AT1 (PF00583) and C-terminal BrD (PF00439) are conserved domains of GCN5-like members; N-terminal ELP (IPR006638) and C-terminal AT1 are domains of ELP3-like; N-terminal Hat1_N (PF10394) and C-terminal AT1 are motifs of HAT1-like members while the only AT1 domain is of HPA2-like proteins. Overlapping domains are hyphenated and represented with a continuous dotted line.

The tomato genome encodes 26 proteins showing similarity to the HAG group (Figure 1). One protein (SlHAG14) was found related to the ELP3 family and one (SlHAG4) to the HAT1 family. SlHAG4, in addition to the AT1 domain, also has a MOZ_SAS motif (PF01853) that is typical of MYST acetyltransferases (HAMs) [35]. To date, this combination of domains was never reported. As regards the GCN5 family, no member appears to have been revealed in tomato by preliminary BLAST interrogation. However, a domain-based search allowed us to identify SlHAG1, carrying a C-terminal BrD domain (PF00439) at the 3^′^-end of Solyc10g045400, as a member of this family. Two other proteins, Solyc02g092260 (SlNAGS1) and Solyc03g043950 (SlNAGS2), identified by domain analysis as plant HAGs, are unlikely histone acetylases. In fact, in addition to AT1 they have an AAK domain (PF00696) that characterizes proteins involved in aminoacid synthesis [36]. Interestingly, we found another family corresponding to HPA2-like HAGs thought previously to be specific to fungi [6]. The tomato HPA2 family includes most HAGs, namely 23 members, SlHAG2, SlHAG3, SlHAG5 to SlHAG13, and SlHAG15 to SlHAG26.

In order to infer the phylogenic history of tomato HAGs, we compared them with Arabidopsis, maize and rice orthologs. HAGs are distributed in six main clades with high bootstrap values (Figure 1), three of which include monocots and dicots while the other three include only dicots. Each clade contains one tomato HAG family, except HPA2 whose members are split into two clades. Interestingly, a subclade of HPA2 members includes eight genes (*SlHAG11*, *SlHAG19-22*, *SlHAG24-26*) that are all closely localized on chromosome 8 in a cluster of about 82 Kb. This finding suggests that the ancestral locus experienced a series of tandem duplication events.

The existence of so many HAG members in the tomato proteome compared with Arabidopsis as well as monocots led us to investigate HAGs in Arabidopsis in greater depth. BLAST search using the AT1 domain as a query returned 33 proteins in Arabidopsis, thereby giving a number close to tomato. Based on the domain composition, in Arabidopsis we identified At2g22910 and At4g37670 which in addition to AT1 have the AAK domain (PF00696). Similarly to tomato, it is likely that these two proteins are not histone acetylases. Phylogenetic analysis of tomato and Arabidopsis HAGs indicates that the different subgroups evolved differently in these species (see Additional file 1). For example, gene duplication events giving rise to the subgroup including SlHAG19 to SlHAG26 likely occurred only in tomato while the orthogroup that comprises SlHAG6 appears to have experienced an expansion only in Arabidopsis.

As mentioned above, SlHAG4 is a peculiar HAG, having both the typical HAT1_N domain and an MOZ_SAS domain. In order to understand the origin of this combination of domains, we performed extensive research through Interpro (http://www.ebi.ac.uk/interpro) into the genomes of fully sequenced organisms (http://www.ncbi.nlm.nih.gov/sites/genome) and particularly in plants (http://www.phytozome.org). Intriguingly, a domain structure similar to that of SlHAG4 was found mostly in plants and additionally in the brown alga *Ectocarpus siliculosus* (Chromoalveolata) and in *Trichoplax adhaerens* (Animalia). The existence of SlHAG4-like proteins in different organisms suggests that histone acetylases with both HAT1_N and MOZ_SAS domains can be categorized as members of a new family which we name GNAT/MYST-Like (GML).

Additional file 2 shows the proteins with the highest similarity to SlHAG4. Out of 32 species belonging to Plantae, 12 evidenced proteins with both HAT1_N and MOZ_SAS domains. Interestingly, these species are not randomly distributed among the different orders. Indeed, GML proteins seem to be lacking in *Brassicales, Poales, Ranunculales* and *Volvocales*, although the scant sequence data suggest caution regarding this finding. The distribution of GML proteins in Planta, Animalia and in Chromoalveolata suggests that the combined domains AT1 and MOZ_SAS occurred early on in evolutionary history. However, most of the organisms show HAT1_N and MOZ_SAS domains in two functional distinct families, GNAT and MYST histone acetylases, respectively. Due to lack of information about the biological function of GML proteins, we can only speculate that the separation of the two domains could confer an advantage for nuanced control of the histone acetylation level in the genome.

**Figure 2:**
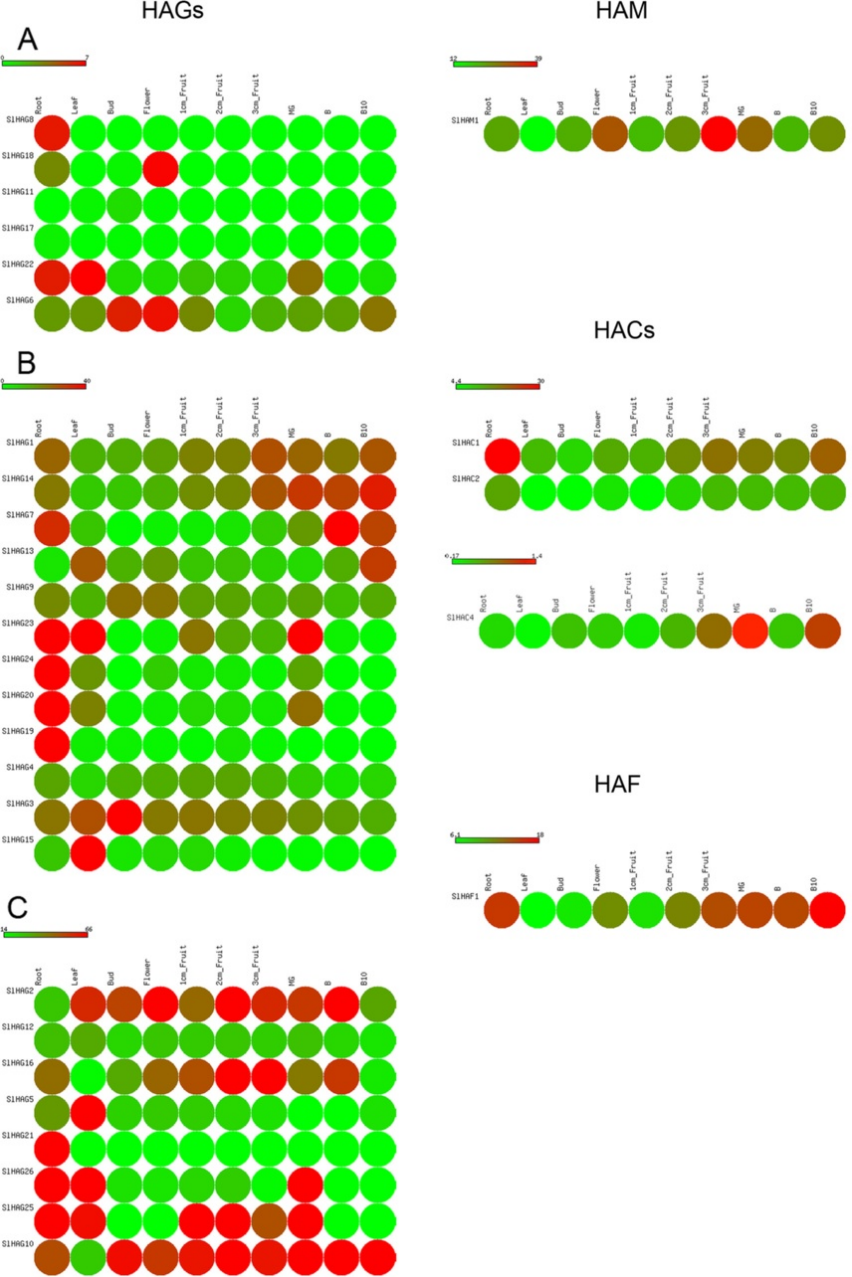
**Expression profiles of tomato*HATs.*** Heat map of RNA-seq expression data from root, leaf, bud, flower, 1cm_fruit, 2cm_fruit, 3cm_fruit, mature green fruit (MG), berry at breaker stage (B) and berry ten days after breaking (B10). *HAGs* with low, middle and high expression values are reported in **A**, **B** and **C**, respectively. The expression values are measured as reads per kilobase of exon model per million mapped reads (RPKM).

To address the question of the possible function of tomato *HAGs* we examined their expression profiles in several organs (Figure 2). Given the wide range of expression values, we categorized the tomato *HAGs* in three groups of low (Figure 2A), middle (Figure 2B) and high expression (Figure 2C). Among low-expressed members, *SlHAG11* and *SlHAG17* did not show any preferential expression in the analyzed organs as compared to the other members that might have a different function. *SlHAG8* and *SlHAG22* could play a role in vegetative development, and by contrast *SlHAG18* and *SlHAG6* in reproductive development. The middle-expressed group of genes evidenced broad-ranging activities, except *SlHAG15* and *SlHAG19* which are strongly expressed in leaves and roots, respectively. The expression profiles in the group of high-expressed members suggest a wide functional role for some *HAGs* (*SlHAG2*, *SlHAG16*, *SlHAG10*, and *SlHAG25*) in contrast to *SlHAG5* and *SlHAG21* preferentially expressed in roots and leaves, respectively. The Arabidopsis genome was predicted to encode three HAGs, AtHAG1, AtHAG2 and AtHAG3*,* which belong to GCN5, HAT1 and ELP3 families, respectively [6]. Interestingly, HPA2-like HAGs also occur in Arabidopsis and one member of this family (AtMCC1) was recently found [37]. In tomato, the closest homologs of AtHAG1/AtGCN5, AtHAG3 and AtMCC1 are SlHAG1, SlHAG14 and SlHAG12, respectively. Such proteins are likely to accomplish specific functions in tomato as they do in Arabidopsis since their genes show comparable expression profiles in similar organs, except for *AtHAG1/AtGCN5*. In particular, *AtHAG1* plays an essential role in many plant development processes, such as meristem function, cell differentiation, leaf and floral organogenesis, and responses to light and cold [38]. *AtHAG3* is involved in transcription elongation, cell proliferation, leaf axis development, seedling and root growth [39-41], and *AtMCC1* was shown to be involved in flowering time and meiosis [37].

#### HAMs

The tomato proteome has one MYST acetyltransferase, namely SlHAM1, that is a 477-aa long protein characterized by N-terminal Chromo (PF00385), C2H2 (PF00096), and C-terminal MOZ_SAS (PF01853) domains, that are typical of class I HAMs [35]. Previous studies have shown that other plant HAMs belong only to the class I [35]. Phylogenetic analysis (see Additional file 3) shows that HAMs are distributed in two clades, one of which includes tomato as well as Arabidopsis proteins. The other clade contains two proteins from monocots, maize and rice. This separation indicates that a single ancestral *HAM* gene gave rise to HAMs in monocots and dicots, being a specific event of duplication at the origin of the expansion of this family in Arabidopsis and maize. The expression pattern of *SlHAM1* shows that it is expressed in all the examined organs with the highest expression in flowers and in 3 cm fruit (Figure 2). Latrasse and colleagues [35] found that *AtHAM1* and *AtHAM2* are strongly expressed in flowers and act redundantly in male and female gametophyte development. This evidence suggests that *SlHAM1*, in addition to its putative role in seed and/or fruit development, could play a role in gametogenesis like the Arabidopsis ortholog.

#### HACs

**Figure 3:**
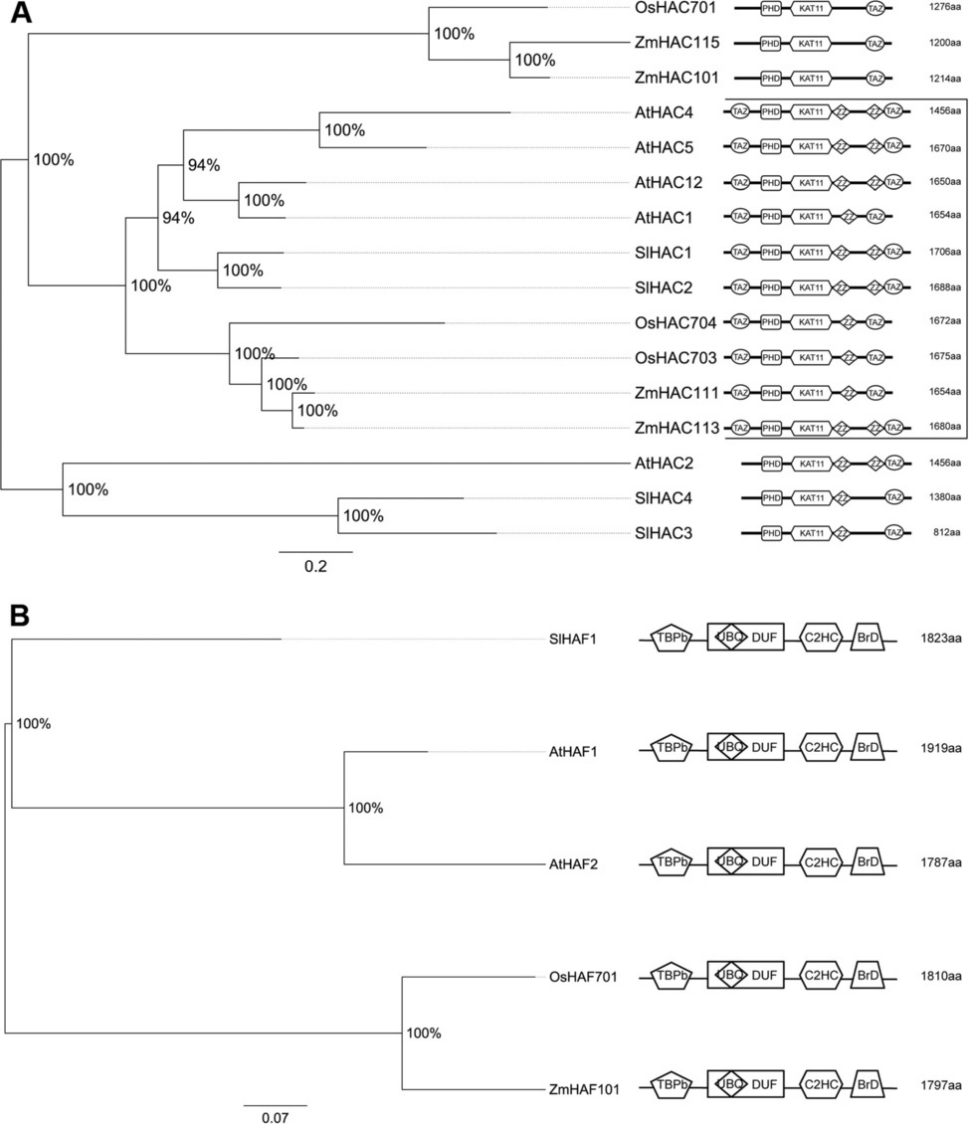
**Phylogenetic tree and domain composition of HAC and HAF proteins.** Bayesian phylogenetic tree of HAC (**A**) and HAF (**B**) predicted proteins from *Arabidopsis thaliana* (At), *Oryza sativa* (Os), *Solanum lycopersicon* (Sl) and *Zea mays* (Zm). Bootstrap values higher than 50% are shown. The tree is drawn to scale, with branch lengths measured in the number of substitutions per site. (**A**) KAT11 (PF08214), PHD-finger (PF00628) and zf-TAZ (PF02135) are conserved domains of HAC proteins. (**B**) N-terminal kinase (PF09247) (TBPb), ubiquitin (PF00240), zinc-finger C2HC (PF01530), and C-terminal bromo BrD (PF00439) are conserved domains of HAFs. A domain of unknown function DUF3591 is also shown.

The present survey identified four proteins belonging to the HAC group in tomato (SlHAC1 to SlHAC4). As shown in Figure 3A, the domain composition of HACs is variable but all share the typical domains of this class [6]. Tomato HACs are included together with Arabidopsis HACs in two main clades separated from the clade containing HACs of rice and maize lacking the ZZ-domain (Figure 3A). In the most expanded clade (boxed in Figure 3), the dicots form a distinct group compared with monocots. Overall, data suggest that different gene duplication events gave rise first to the two groups of HACs both in monocots and dicots, and subsequently to the expansion of this family in both phyla. Interestingly, the expansion was slightly larger in Arabidopsis than in tomato.

In order to gain insight into the possible role of tomato *HACs*, we examined their expression profiles in different tomato organs (Figure 2). *SlHAC4* shows the strongest expression in fruit at different developmental stages. It is interesting that the peak of *SlHAC4* expression occurs in mature green berries and is followed by a strong reduction in fruit at breaker stage, thereby suggesting a role in the transition between these two fruit developmental stages. *SlHAC1* and *SlHAC2*, forming a distinct clade, are the most widely expressed tomato *HACs*, with the latter showing lower expression values. Similarity between SlHAC1 and SlHAC2 in terms of sequence and expression profile in reproductive organs suggests a functional redundancy that is analogously reported for Arabidopsis homologs *AtHAC1/AtHAC5* and *AtHAC1/AtHAC12*[42]. The presence of SlHAC1 and SlHAC2 in the same clade of Arabidopsis AtHAC1, AtHAC5 and AtHAC12 further supports a role of these proteins in tomato reproduction. Indeed, knockdown of *AtHAC1* induced reduced fertility and late flowering [43] and analysis of *hac1*/*hac5* and *hac1*/*hac12* double mutants highlighted their role in flowering time in Arabidopsis [42]. *SlHAC3* is likely a pseudogene since it does not appear to be expressed in the tissues under analysis.

#### HAFs

Tomato proteome has one TAF_II_250 protein (SlHAF1) (Figure 3B) that shows the same domain composition of Arabidopsis*,* rice and maize HAFs [6]. Phylogenetic comparison with these species evidenced that SlHAF1 forms a distinct clade with AtHAF1 and AtHAF2 separated from OsHAF701 and ZmHAF101 (Figure 3B). Interestingly, *SlHAF1*, albeit expressed in all the organs considered, has the strongest expression in roots and in fruit, particularly in berries ten days after breaking, thereby suggesting an important role in fruit maturation (Figure 2).

### Tomato HDACs

#### HDAs

**Figure 4:**
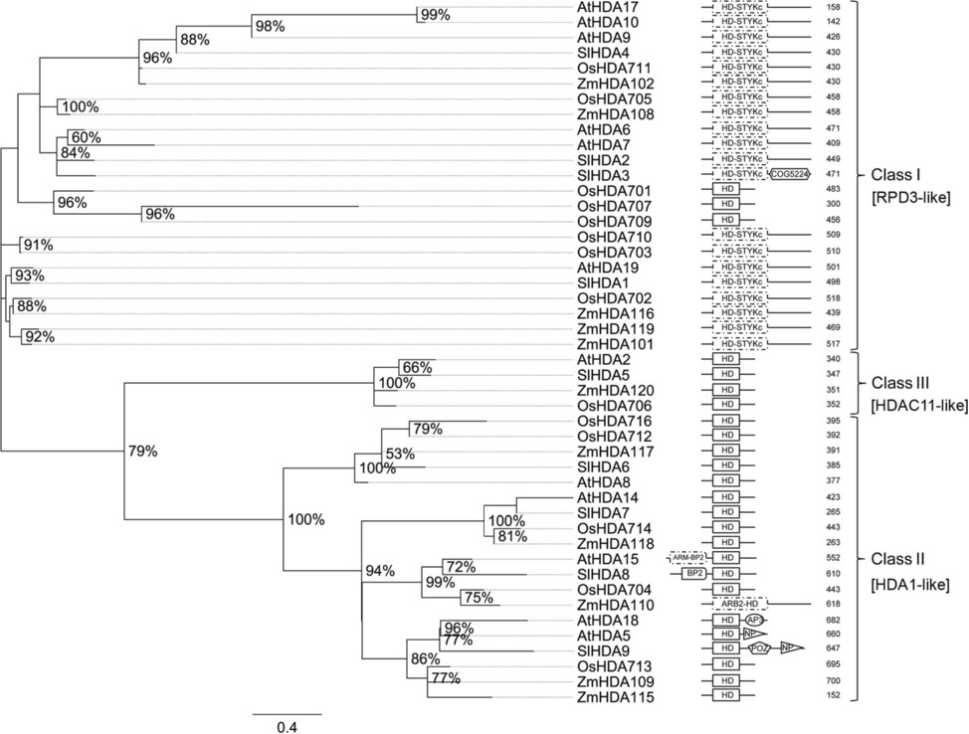
**Phylogenetic tree and domain composition of HDA proteins.** Maximum likelihood phylogenetic tree of HDA predicted proteins from *Arabidopsis thaliana* (At), *Oryza sativa* (Os), *Solanum lycopersicon* (Sl) and *Zea mays* (Zm). Bootstrap values higher than 50% are shown. The tree is drawn to scale, with branch lengths measured in the number of substitutions per site. Class I and Class II include the homologs of RPD3 and of HDA1 from yeast, respectively; Class III includes the homologs of human HDAC11. The hist_deacetyl domain (PF00850) is the conserved domain of HDA proteins.

Investigation of the tomato proteome revealed nine RPD3/HDA1 family members. The phylogeny of tomato HDAs evidences that they cluster with HDAs of Arabidopsis, maize and rice (Figure 4) in accordance with the subdivision of this family into three classes as reported in the literature [6,44]. This family had a higher expansion in monocots, especially in rice, than in dicots where Arabidopsis has the highest number. In addition to the Hist_deacetyl domain (PF00850), new conserved domains were found in tomato HDAs as well as in orthologs of Arabidopsis, rice and maize. Indeed, as shown in Figure 4, Class I SlHDAs have an STYKc domain (SM00221), and a Ser/Thr/Tyr kinase catalytic domain, overlapping with the Hist_deacetyl domain. Moreover, a C-terminal COG5224 domain, which is involved in DNA-binding, is found in SlHDA3. As regards Class II, a zf-RanBP domain (PF00641), which binds Ran-GDP involved in nuclear transport, occurred in SlHDA8 and in SlHDA9, and a C-terminal nucleoside phosphorylase domain (NP) together with a POZ domain (PF00651) was found. The presence of the POZ domain, which is a homo/heterodimerizing domain evidenced in histone deacetylase-containing complexes, suggests that SlHDA9 could take part in a multi-protein complex. The occurrence of BP and NP domains as well as a new domain arrangement (AP3) was also evidenced in Arabidopsis HDAs.

In order to understand the candidate function of tomato *HDAs*, we looked at their expression profiles (see Additional file 4). Given the wide range of expression values, we categorized the tomato *HDAs* in three groups having low (see Additional file 4A), middle (see Additional file 4B) and high expression (see Additional file 4C). *SlHDA2* expressed mostly in root and bud is the lowest expressed gene among the tomato *HDAs*. Its expression profile suggests a role in highly dividing tissues such as root and flower meristems. The middle-expressed *HDA* members show very different expression profiles. Among them, *SlHDA9* could exert a possible role in root development as supported by its strong expression in this organ and by its similarity to AtHDA5 and AtHDA18 [45]. A complementary role of *SlHDA5*, *SlHDA6* and *SlHDA7* in fruit development from 1 cm to B10 stage is suggested by their peaks of expression in these stages. Finally, the highly expressed *SlHDA1* and *SlHDA3* show the strongest expression at B10 and B fruit stages, respectively, thereby supporting a possible role in tomato fruit ripening. SlHDA1 and SlHDA3 have respectively a sequence similarity with AtHDA6 and AtHDA19 that in Arabidopsis have been linked to flowering, embryo development and other biological processes [11,46,47].

#### SRTs

In the tomato proteome, we identified two histone deacetylases belonging to the SIR2 family, namely SlSRT1 and SlSRT2 (see Additional file 5). They are characterized by an SIR2 domain (PF02146) and correspond to LeSRT1105 and LeSRT1104, previously described by Pandey and colleagues [6]. The expression profiles of tomato *SRT* genes evidence expression peaks of *SlSRT1* in bud and in 1 cm-sized fruit while *SlSRT2* was expressed in flower and in fruit at B10 (see Additional file 4). These findings suggest that *SlSRT1* could play a role in the early stages of fruit development as well as in early gamete development whereas *SlSRT2* is involved later in both fruit ripening and in gametogenesis. The expression profile of *SlSRT2* also supports a role in *FLC* regulation as suggested for Arabidopsis counterparts by Bond and colleagues [48].

#### HDTs

According to the results of Pandey and colleagues [6] who described three HDTs in tomato proteome (HDT1101, HDT1102, HDT1103) we found SlHDT1, SlHDT2 and SlHDT3 corresponding to HDT1102, HDT1103 and HDT1101, respectively (see Additional file 6). SlHDT2 shows a C-terminal zinc finger domain in addition to the predicted HD2 domain (EFWG motif at the N-terminus). The evolutionary history of plant HDTs, including those of tomato, was well illustrated by Pandey and colleagues [6]. As shown in Additional file 4, the preferential expression of tomato *HDTs* occurs at early stages of fruit development. In particular, *SlHDT1* is highly expressed in 1 cm fruit, *SlHDT2* in both 1 cm- and 3 cm-sized fruits, *SlHDT3* in 3 cm fruit and in mature green berries. Overall, these expression profiles suggest a role of tomato *HDTs* in fruit development. Interestingly, tomato HDTs seem to be all closely related to AtHDT3 (see Additional file 6) that was shown to be involved in ABA response and seed germination [45].

### Tomato HMTs

#### SDGs

**Figure 5:**
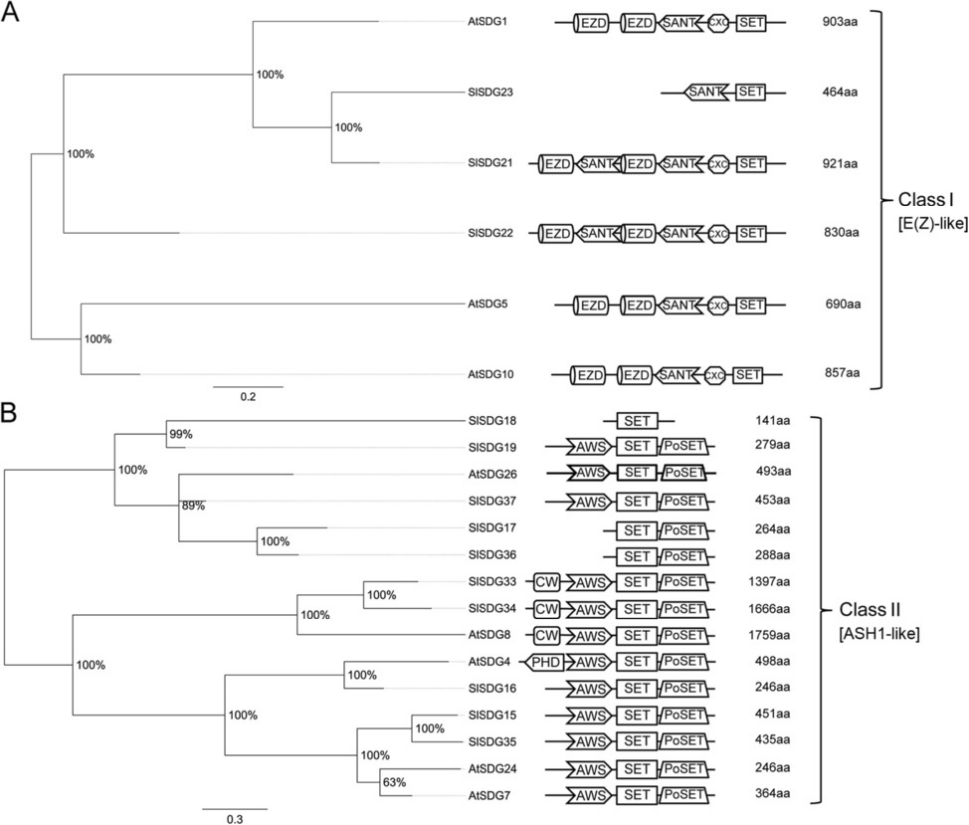
**Phylogenetic trees and domain composition of Class I and Class II SDG proteins.** Maximum likelihood phylogenetic trees of Class I (**A**) and Class II (**B**) SDG predicted proteins from *Arabidopsis thaliana* (At) and *Solanum lycopersicon* (Sl). Bootstrap values higher than 50% are shown. The tree is drawn to scale, with branch lengths measured in the number of substitutions per site. Two EZD, SANT (SM00717), CXC (PF03638), and SET (PF00856) are conserved domains of Class I. N-terminal AWS (SM00570), SET and Post-SET (SM00508) are conserved domains of Class II. The PHD domain (PF00628) and CW domain (SM00605) are also found in Class II proteins.

**Figure 6:**
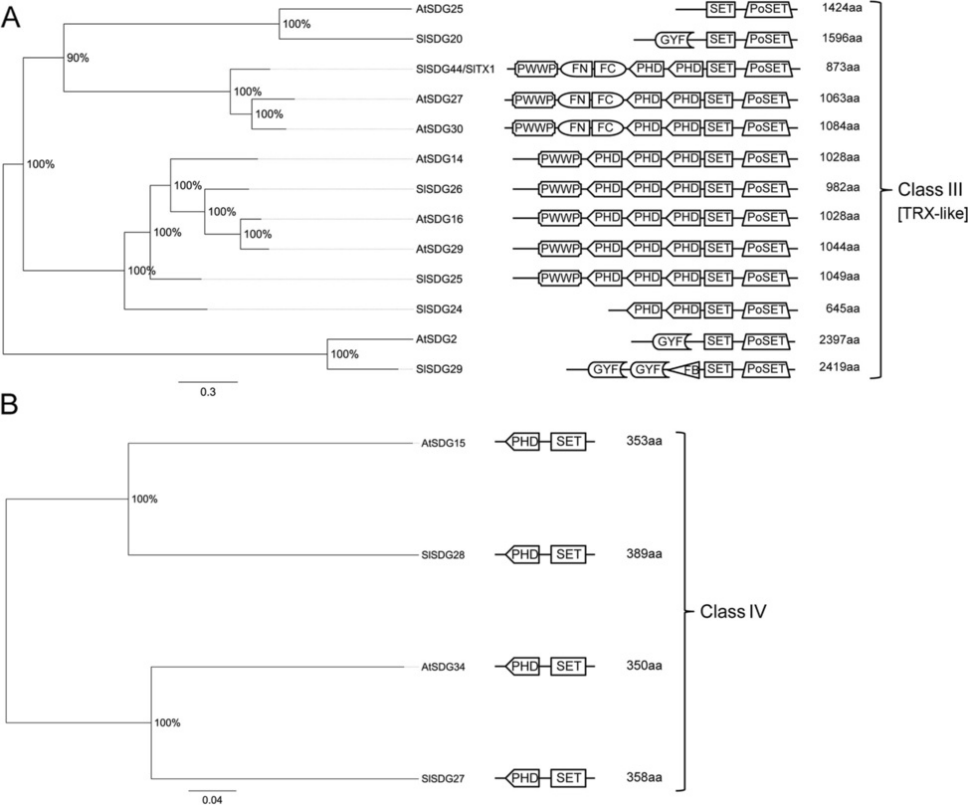
**Phylogenetic trees and domain composition of Class III and Class IV SDG proteins.** Maximum likelihood phylogenetic trees of Class III (**A**) and Class IV (**B**) SDG predicted proteins from *Arabidopsis thaliana* (At) and *Solanum lycopersicon* (Sl). Bootstrap values higher than 50% are shown. The tree is drawn to scale, with branch lengths measured in the number of substitutions per site. N-terminal PWWP (PF00855), FYRN (PF05964), FYRC (PF05965), two PHD, SET, and Post-SET are conserved domains of Class III. Some Class III proteins lack the FYRN (PF05964) and FYRC (PF05965) domains. Other Class III have N-terminal GYF (PF02213), SET and Post-SET domains. N-terminal PHD and C-terminal SET are conserved domains of Class IV.

**Figure 7:**
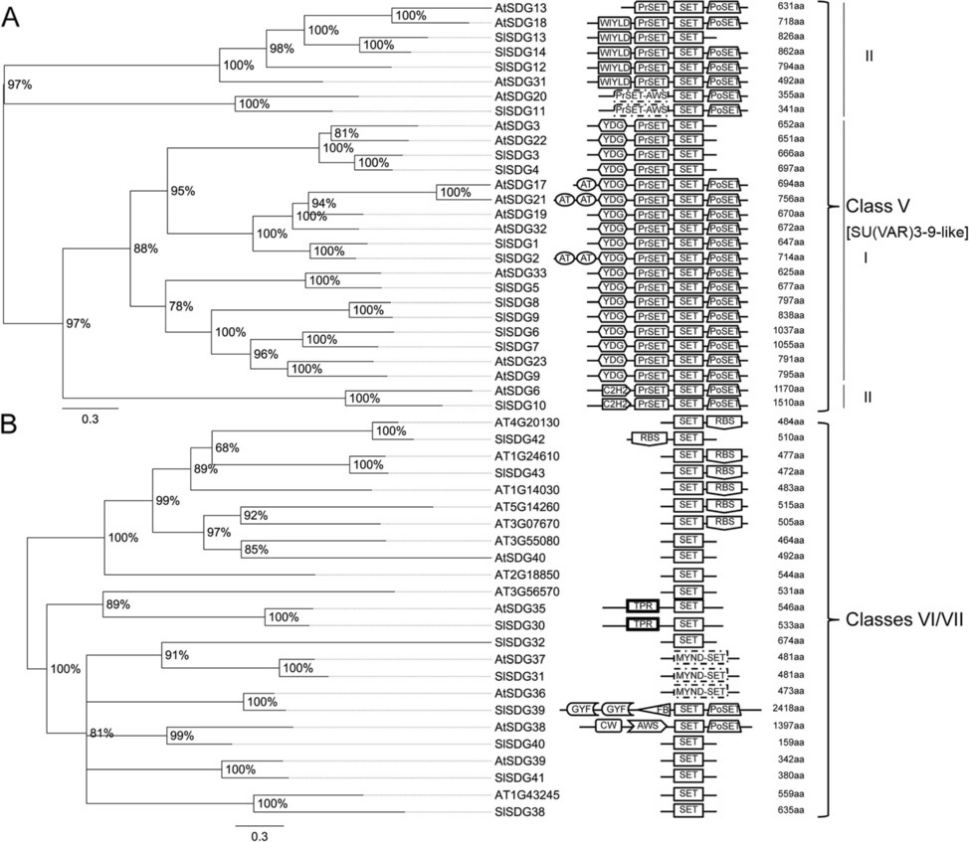
**Phylogenetic trees and domain composition of Class V and Class VI/VII SDG proteins.** Maximum likelihood phylogenetic trees of class V and class VI/VII SDG predicted proteins from *Arabidopsis thaliana* (At) and *Solanum lycopersicon* (Sl). Bootstrap values higher than 50% are shown. The tree is drawn to scale, with branch lengths measured in the number of substitutions per site. N-terminal SRA-YDG (PF02182), Pre-SET, SET and Post-SET are conserved domains of the group I of Class V; N-terminal WIYLD (PF10440), or C2H2 (PF00096) or absence of domain, Pre-SET, SET and Post-SET are conserved domains of the group II of Class V (so-called SUVH-related). Proteins with an interrupted SET domain or SET-related proteins are indicated as Class VI and VII.

We identified 43 SET-Domain Group (SDG) proteins in tomato belonging to seven classes like Arabidopsis SDGs according to the classification of Springer and colleagues [49] (Figures 5, 6 and 7). In detail, three proteins, SlSDG21, SlDG22 and SlSDG23, clustered with class I AtSDGs (AtSDG1, AtSDG5, AtSDG10) that are homologous to E(z) (Figure 5A). Although SlSDG21 and SlSDG22 show similar domain architecture to Arabidopsis class I SDGs, they have an additional SANT domain, while SlDG23 has lost the two conserved EZDs (enhancer of zeste domains). Ten proteins, SlSDG15 to SlSDG19 and SlSDG33 to SlSDG37, cluster with five Arabidopsis proteins annotated as homologs to ASH1 (class II) (Figure 5B). The expansion of this class in tomato likely arose from gene duplications generating also pseudogenes (see below). Tomato SlSDG15, SlSDG16, SlSDG19, SlSDG33 to SlSDG35, SlSDG37 show a domain arrangement similar to Arabidopsis members while SDG17, SDG18, and SDG36 lack conserved domains of this class. Six proteins (SlSDG20, SlSDG24-26, SlSDG29, SlSDG44, previously described as SlTX1 by Sadder et al. [50]) belong to class III of SDGs (Figure 6A), being homologous to TRITHORAX (TRX). They have the same domain architecture (SlSDG44, SlSDG25-26) as their Arabidopsis counterparts or a GYF and F-box (SlSDG29) in addition to the SET and Post-SET domains. Moreover, we found that tomato as well as Arabidopsis has proteins with three PHD domains, contrasting with findings previously reported in Arabidopsis [49]. SlSDG24 has two PHD domains but one seems to be truncated at the N-terminus because it lacks the PWWP domain. Two TRX-related proteins (SlSDG27 and SlSDG28) belong to class IV SDGs (Figure 6B). This class includes proteins only present in yeast and plants [49]. Fourteen tomato SDGs (SlSDG1 to SlSDG14) belong to class V (Figure 7A). These are homologous to SU(VAR)3-9 and are distributed in two main clades containing members of the first or second subgroup of this class [49]. Some members lack the Post-SET domain and others gain AT-hook domains as the closest Arabidopsis orthologs. Seven members (SlSDG30-32, SlSDG38-43) cluster within class VI and two within class VII of SDGs (Figure 7B). These classes include proteins with an interrupted SET domain or SET-related proteins. The domain architecture of tomato and Arabidopsis members belonging to these classes is quite similar, except SlSDG39 which shows a domain composition typical of Class III SDGs.

**Figure 8:**
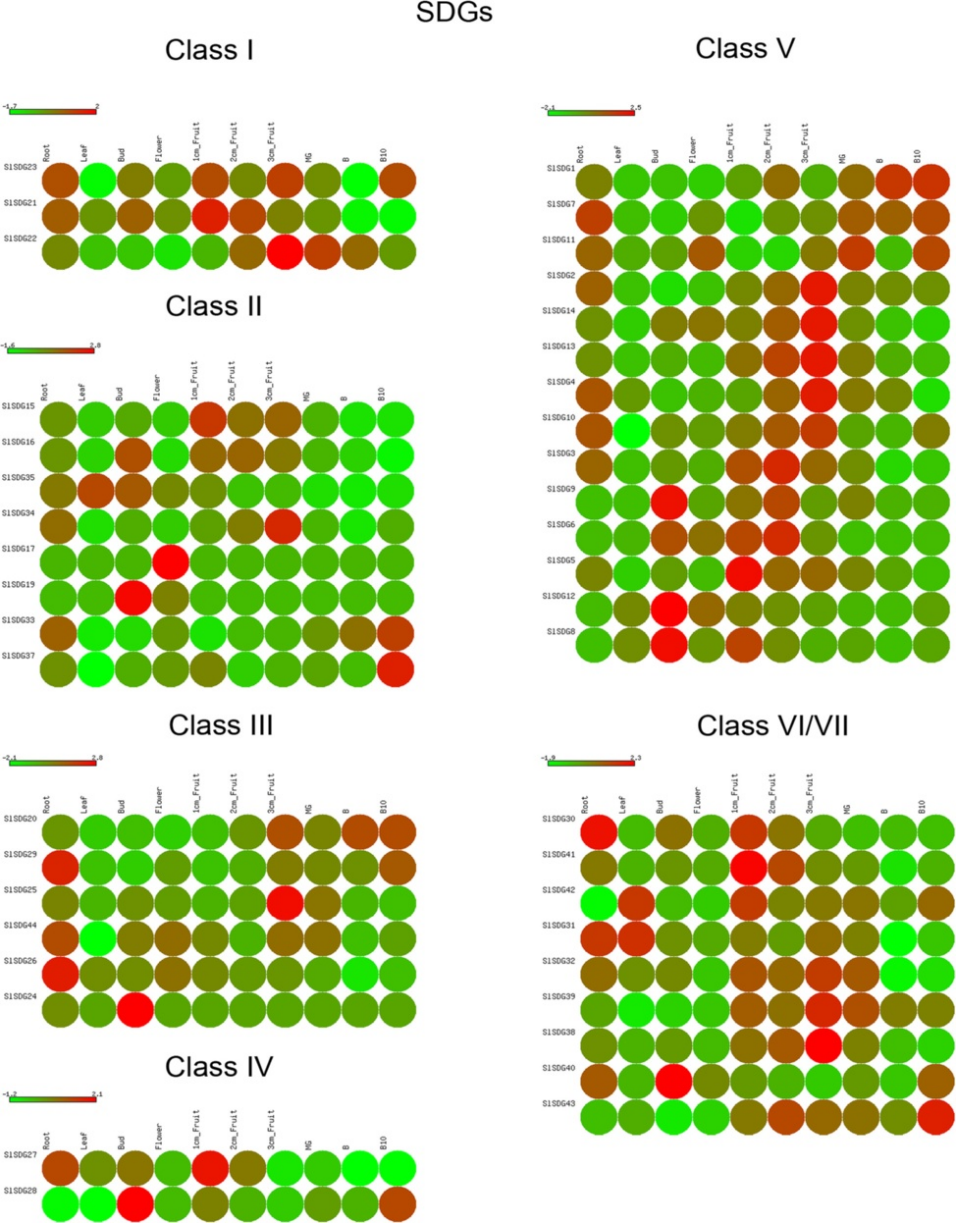
**Expression profiles of tomato*SDGs.*** Heat map of RNA-seq expression data from root, leaf, bud, flower, 1cm_fruit, 2cm_fruit, 3cm_fruit, mature green fruit (MG), berry at breaker stage (B) and berry ten days after breaking (B10). The expression values are measured as reads per kilobase of the exon model per million mapped reads (RPKM).

In order to gain insights into the biological role of tomato *SDGs*, we analyzed their expression profiles by grouping *SDGs* according to their class (Figure 8). *SlSDG23* and *SlSDG21* (Class I) have similar expression profiles, being mainly expressed in root, bud and fruit up to 3 cm, while *SlSDG22* is mostly expressed in 2 cm fruit up to B stages. On the basis of these expression profiles we could argue that the first two genes play redundant roles in root and fruit development and *SlSDG22* is likely to be more specific to the later stages of fruit maturation.

As regards class II *SDGs*, some genes with very specific peaks of expression may be noted. Indeed, *SlSDG35* is strongly expressed in leaves, *SlSDG34* in fruit at the 3 cm stage, *SlSDG17* in flowers, and *SlSDG19* in buds. These expression profiles suggest a possible wide subfunctionalization of class II *SDGs* in tomato with a low degree of redundancy. A role in fruit development could be played by *SlSDG33* with an expression profile similar to *AtSDG8*[51] which regulates gene expression in the carotenoid pathway [52]. *SlSDG16* is mostly expressed in buds and in the early stages of fruit development. Interestingly, this gene could share some functions with its Arabidopsis counterpart, *AtSDG4*. Indeed, the latter mainly expressed in pollen is involved in pollen tube growth and reproduction in Arabidopsis [53]. *SlSDG18* and *SlSDG36* were noticed to behave like pseudogenes, not being expressed in any of the organs analyzed.

Among the class III *SDGs*, *SlSDG29*, *SlSDG44* and *SlSDG26* could play redundant roles in root development, as could *SlSDG20* and *SlSDG29* in fruit maturation. The latter is closely related to AtSDG2 which was shown to affect vegetative growth and reproduction in Arabidopsis by regulating the expression of hundreds of genes [54,55]. Moreover, the expression profile of *AtSDG2*[51] is similar to that of *SlSDG29* in comparable organs, thereby supporting the idea of similar functions. SlSDG44 is related to AtSDG27 that was shown to regulate the expression of a xyloglucanase [56] which belongs to a class of enzymes involved in tomato fruit ripening [57]. In a similar fashion, *SlSDG44* could act in fruit ripening since it is highly expressed in fruits, particularly at the MG stage. On the contrary, some degree of functional divergence seems to have occurred between SlSDG20 and its homolog AtSDG25 involved in flowering time in Arabidopsis [58]. Indeed, the latter is more expressed in flowers while *SlSDG20* is very poorly expressed in this organ.

The two members of tomato Class IV *SDGs* have different expression profiles: while *SlSDG27* is strongly expressed in roots and fruit at the 1 cm stage, *SlSDG28* is mostly expressed in buds and in fruit at the B10 stage. These differences suggest that the two genes evolved different functions, with *SlSDG28* being mainly involved in reproduction.

Tomato *SDGs* of class V may show a high degree of redundancy in some functions. Indeed, *SlSDG3*, *SlSDG9*, *SlSDG6* and *SlSDG5* have their highest expression in fruit at 1 cm and 2 cm, *SlSDG2*, *SlSDG14*, *SlSDG13*, *SlSDG4* and *SlSDG10* have their peak expression in fruit at the 3 cm stage, while *SlSDG1* and *SlSDG7* are particularly expressed in fruit at MG up to B10 stages. Therefore, these expression profiles suggest that they might play roles in fruit and/or seed at sequential stages of development. Moreover, *SlSDG9* is highly expressed in buds as well as *SlSDG12* and *SlSDG8*, suggesting that they could have a function in meiosis or in flower development.

As with the above-reported *SDGs*, also the members of classes VI and VII may have a possible redundant function. For example, *SlSDG30*, *SlSDG41* and *SlSDG42* are highly expressed in 1 cm fruit while *SlSDG32*, *SlSDG39* and *SlSDG38* in 3 cm fruit, thereby suggesting sequential functions in embryo/fruit development. A more specific expression profile is shown by *SlSDG40* which evidenced peak expression in the bud, indicating a role in gamete and/or flower development. However, the putative involvement of these genes in development has not been investigated in any plant species.

#### PRMTs

We identified nine PRMTs in tomato (SlPRMT1 to SlPRMT9). SlPRMT8 was already described by Krause and colleagues [59] and was named PAM1.1. Specific patterns in the catalytic AdoMet_Mtase domain (CD02440) [59] allowed us to categorize SlPRMT1, 2, 3, 5, 7, and 9 as class I PRMTs while SlPRMT4 and SlPRMT6 belong to class II (see Additional file 7). For class I some duplication events were highlighted in dicot species.

The expression profiles of tomato *PRMTs* (see Additional file 8) suggest functional redundancy among these genes since some organs are characterized by two or more *PRMTs* with high expression levels. This is the case of roots where *SlPRMT9*, *SlPRMT7* and *SlPRMT3* have their relative strongest expression. *SlPRMT8*, *SlPRMT2*, and *SlPRMT5* were expressed in fruit at the 1 cm stage while *SlPRMT4* and *SlPRMT1* at the B10 stage. To investigate the biological function of *SlPRMTs*, we considered the role of orthogroups in Arabidopsis*.* SlPRMT5 and SlPRMT8 belong to the same clade as AtPRMT11 and AtPRMT12. The latter were suggested to be in the same histone methylation complex on the basis of their physical interaction [60] and spatial expression profiles. By contrast, *SlPRMT5* and *SlPRMT8* have quite different expression profiles and, when similar organs are compared between the two species, only the first has a profile resembling that of Arabidopsis counterparts. On this basis, we hypothesize that SlPRMT5 and SlPRMT8 evolved independent functions, with SlPRMT5 perhaps retaining the biological role of AtPRMT11 and AtPRMT12. If this is true, SlPRMT5 should be involved in flowering time, flower morphology and fertility as well as in leaf development [61]. SlPRMT7 is the closest homolog to AtPRMT10 which was shown to be a component in the autonomous pathway which controls the floral transition in an FLC-dependent manner [62]. Since the expression profiles of these two genes are comparable, *SlPRMT7* might also have a functional role in flowering time. SlPRMT3 and SlPRMT9 grouped with AtPRMT13 and AtPRMT14 that were shown to redundantly control the floral transition [63]. Accordingly to their functional redundancy, *AtPRMT13* and *AtPRMT14* have very similar expression profiles. On the other hand, *SlPRMT3* and *SlPRMT9* differ greatly in expression profile, also vis-à-vis their Arabidopsis counterparts, when similar organs are compared [51]. *SlPRMT3* and *SlPRMT9* could play different roles in tomato development and might not be involved in flowering time. SlPRMT6 is the closest homolog to AtPRMT5, which was shown to be involved in vegetative growth and flowering time [64,65]. The different expression profiles of *AtPRMT5*[51] and *SlPRMT6* suggest that the latter evolved a different role possibly in fruit maturation as evidenced by its expression peak in fruit at the MG stage.

### Tomato HDMs

#### HDMAs

In tomato, we identified 34 proteins showing similarity to HDMA histone demethylases. All are characterized by the C-terminal Amino_Oxidase domain (AOD) (PF01593) but only six (SlHDMA1 to SlHDMA6) also have the N-terminal SWIRM (PF04433) domain that is conserved in all HDMAs. As shown in Additional file 9, HDMAs proteins are distributed in two main clades, comprising one (SlHDMA6) and five tomato members (SlHDAM1-5). Phylogenetic analysis suggests that four ancestors gave rise to the present number of *HDMAs* in tomato, and accordingly at least two events of gene duplication increased the number of *HDMAs* from four to six. In particular, *SlHDMA1, 2* and *SlHDMA4, 5* could have been arisen from a tandem duplication event as suggested by their close position on chromosome seven (not shown).

The Additional file 10 shows the expression profile of tomato *HDMAs*, divided into three groups with low (A), mild (B) and high (C) expression. *SlHDMA2* is barely detectable in buds and in fruit from 2 cm stage to B, while *SlHDMA5* is mostly expressed in buds and flowers, suggesting a major role of the former gene in fruit development and the latter gene in gamete and/or flower development. *SlHDMA4* and *SlHDMA6* are detectable in all organs, pointing out a possible role for these genes throughout development including reproductive stages. *SlHDMA1* is quite uniformly expressed in all plant organs and *SlHDMA3* has a clear preferential expression in fruit from 2 cm stage to B10. Collectively, these profiles indicate that tomato *HDMAs* could play redundant roles in different aspects of fruit development and *SlHDMA3* could be the major histone demethylase in tomato. Moreover, *SlHDMA3* could play a role both in flowering time and root elongation, as suggested by its expression profile and its sequence similarity to AtHDMA3 [66,67].

#### JMJs

The tomato proteome reveals 20 proteins belonging to the JMJ family of HDMs. On the basis of their domain composition we classified them in five classes that take their names from their human counterparts: JMJC-only, KDM4, JMJD6, KDM5 and KDM3 [68]. The tomato JmjC-only class includes three proteins (SlJMJ10-11, and SlJMJ18), the KDM4 class five proteins (SlJMJ1-5), and KDM5 class four proteins (SlJMJ6-8, and SlJMJ16). The classes JMJ6 (SlJMJ9 and SlJMJ12) and KDM3 (SlJMJ13-15, SlJMJ17, and SlJMJ19-20) include two and six members, respectively.

**Figure 9:**
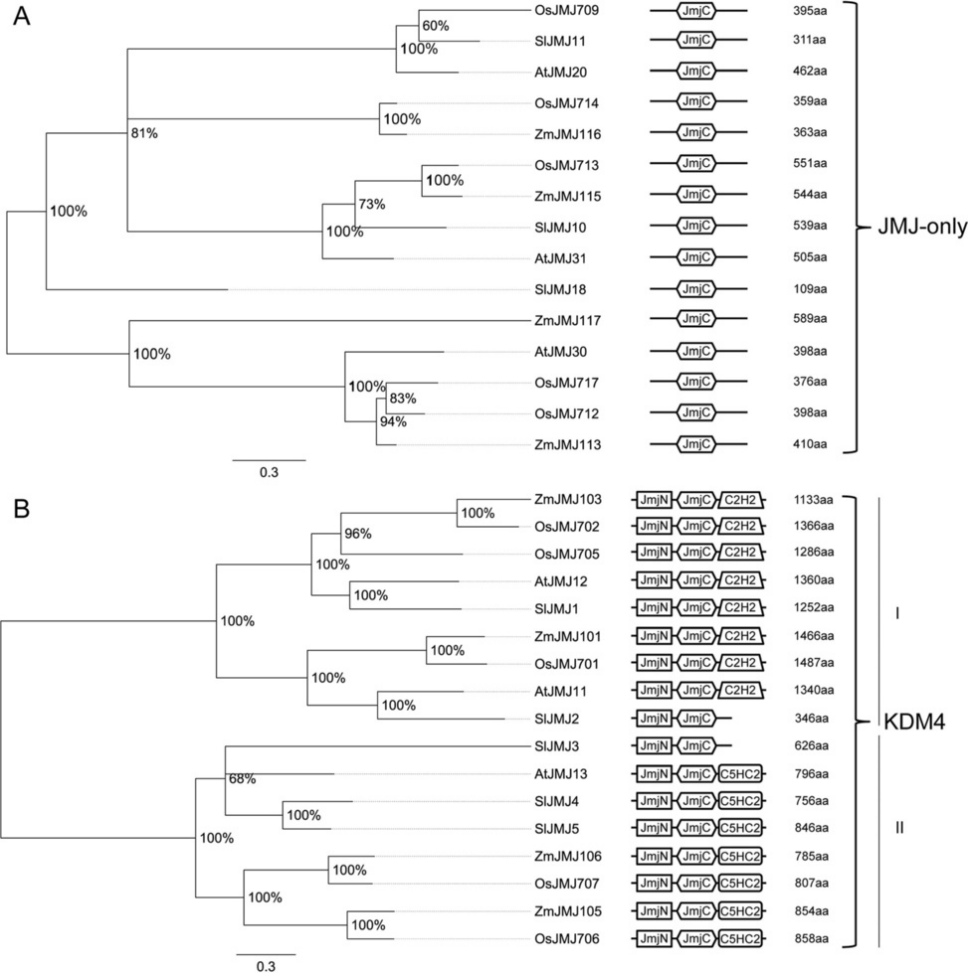
**Phylogenetic tree and domain composition of JMJ-only and KDM4 proteins.** Bayesian phylogenetic tree of JMJ-only (**A**) and KDM4 (**B**) predicted proteins from *Arabidopsis thaliana* (At), *Oryza sativa* (Os), *Solanum lycopersicon* (Sl) and *Zea mays* (Zm). Bootstrap values higher than 50% are shown. The tree is drawn to scale, with branch lengths measured in the number of substitutions per site. JmjC domain (PF02373) is the conserved domain of the JMJs-only class; N-terminal JmjN (PF02375) and JmjC, and C-terminal C5HC2 (PF02928) (subgroup I) or C2H2 (PF00096) (subgroup II) domains are conserved domains of KDM4 proteins.

**Figure 10:**
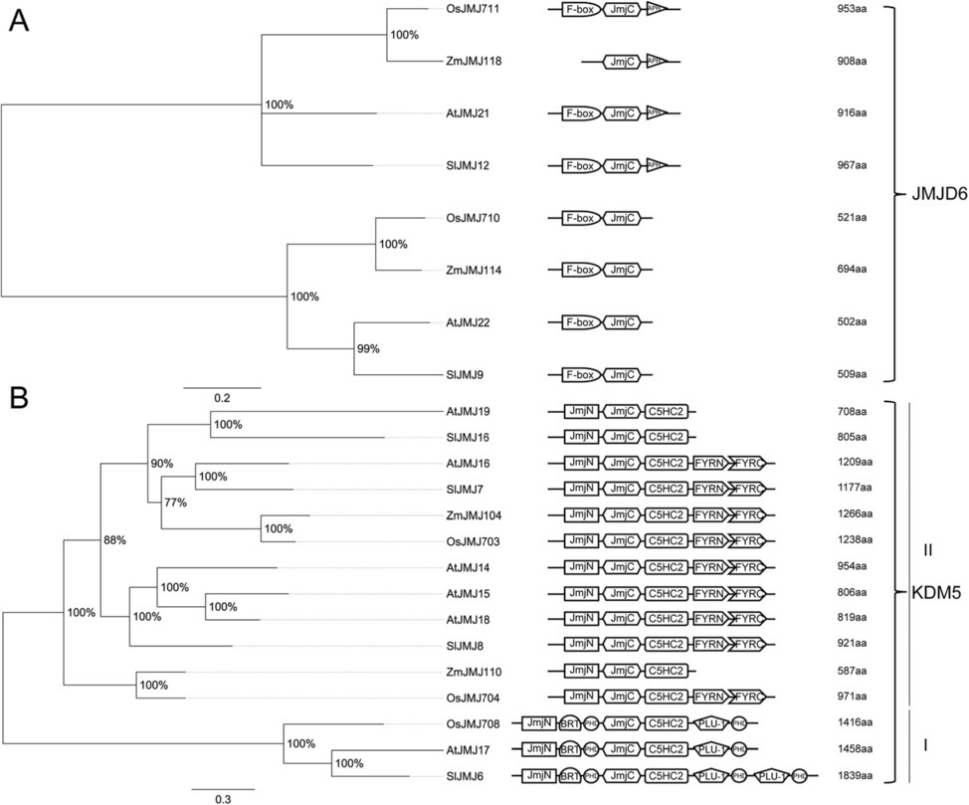
**Phylogenetic tree and domain composition of JMJD6 and KDM5 proteins.** Bayesian phylogenetic tree of JMJD6 (**A**) and KDM5 (**B**) predicted proteins from *Arabidopsis thaliana* (At), *Oryza sativa* (Os), *Solanum lycopersicon* (Sl) and *Zea mays* (Zm). Bootstrap values higher than 50% are shown. The tree is drawn to scale, with branch lengths measured in the number of substitutions per site. N-terminal F-box (PF00646), and C-terminal JmjC are conserved domains of JMJD6s demethylases; JmjN, BRIGHT/ARID (PF01388), two PHD (PF00628), JmjC, and C5HC2 are conserved domains of the subgroup I of KDM5; JmjN, JmJC, C5HC2, FYRN (PF05964) and FYRC (PF05965) are conserved domains of the subgroup II of class KDM5.

The evolutionary history of JMJs was inferred by comparing these proteins in tomato, Arabidopsis, maize and rice (see Additional file 11). Interestingly, a domain-based search led us to identify four new JmjC-only (ZmJMJ113, ZmJMJ115-117), two JMJD6 (ZmJMJ114 and ZmJMJ118) and one KDM3 (ZmJMJ112) proteins in the maize proteome that were absent in the ChromDB and were included in our analysis. As shown in Additional file 11, HDMs are distributed in five main clades, all of which include tomato proteins. Three clades contain exclusively members of classes KDM3, KDM4 and KDM5; the remaining clades contain members belonging both to classes JMJ-only and JMJD6. In the phylogenetic tree (Figure 9A) two main groups of JMJ-only were evidenced, including tomato members in one. This scenario suggests that one ancestor gave rise to the current number of JMJ-only proteins in tomato. All the tomato members included in this class share the same domain architecture with their orthologs. KDM4 class members (Figure 9B) are split into two main clades with two and three tomato proteins. One clade includes the C2HC2-domain proteins and the other the C5HC2 domain proteins [68]. SlJMJ2 and SlJMJ3 did not show the same domain architecture as the other SlKDM4 since the C-terminal domain C2HC2 or C5HC2 is lacking. Two duplication events in tomato as well as in maize and rice expanded the second group, while only an AtJMJ13 is encoded by the Arabidopsis genome. The JMJD6 proteins (Figure 10A) are split into two main clades, each including one tomato protein. The domain architecture of the first group is characterized by the presence of a kinase C-terminal APH domain (PF01636), which is not observed in the other group.

**Figure 11:**
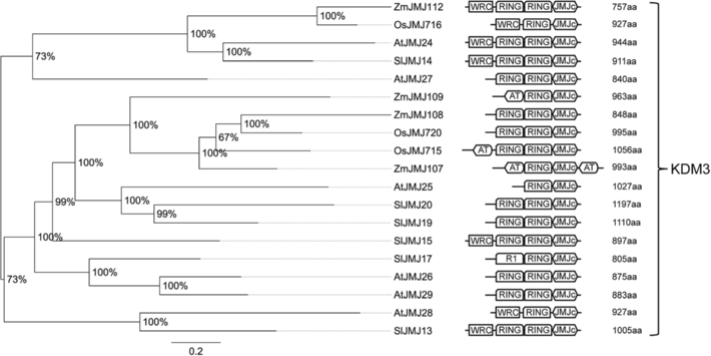
**Phylogenetic tree and domain composition of KDM3 proteins.** Bayesian phylogenetic tree of KDM3 predicted proteins from *Arabidopsis thaliana* (At), *Oryza sativa* (Os), *Solanum lycopersicon* (Sl) and *Zea mays* (Zm). Bootstrap values higher than 50% are shown. The tree is drawn to scale, with branch lengths measured in the number of substitutions per site. RING-finger (IPR001841) and JmjC are conserved domains of KDM3 demethylases.

Phylogenetic analysis of the proteins belonging to this class suggests that they are highly conserved among species. The KDM5 class (Figure 10B) is divided into two main clades, one of which has three tomato proteins, the other only one. The first includes the proteins with the C-terminal FYRN and FYRC domains and the other the BRIGHT/ARID domain proteins [68]. SlJMJ16 lacks the conserved C-terminal domains (FYRN and FYRC) and SlJMJ6 has a duplication of the region encoding the PLU-1 (PF08429)-PHD domains. The PLU-1 domain is involved in the DNA-binding domain and it was not described before in JMJ demethylases. The KDM3 phylogenetic tree (Figure 11) has two main clades with a high bootstrap value. Interestingly, five proteins, three of which are found in tomato (SlJMJ13-15), have an N-terminal WRC domain (PF08879) which includes a putative nuclear localization signal and a zinc-finger motif which was not described previously in this class. A modified RING-finger domain named R1 (PF10497) was also identified in the tomato SlMJ17. A tandem duplication of the *SlJMJ19* gene was observed.

The wide expression profile of tomato *JMJs* (see Additional file 10) in several organs suggests that they could play a global role in plant development. However, some *JMJs* showed specific expression peaks, thereby suggesting particular roles. This is the case of *SlJMJ17* and *SlJMJ7* which are preferentially expressed in roots while *SlJMJ3*, *SlJMJ8*, *SlJMJ4* and *SlJMJ20* in buds and/or flowers, suggesting a role in gamete formation or flower development. Interestingly, SlJMJ8 is the closest homolog to AtJMJ14, which is highly expressed in flowers [51] and acts as a repressor of the photoperiodic pathway [69]. *SlJMJ12*, *SlJMJ16*, *SlJMJ5* and *SlJMJ13* are particularly expressed in fruit at B10, thus suggesting a role in later processes of fruit and/or embryo/seed development.

### Association of tomato HMs to *S. pennellii* introgression lines (ILs): a case study

To identify candidate genes involved in epigenetically regulated processes by means of *in silico* analysis we looked for ILs where HMs were associated, based on their map position on the tomato genome (see Additional file 12). We failed to recover ILs for *SlHAG15*, which is not assigned to any chromosome (chr), and for *SlJMJ4* (chr4), *SlPRMT6* (chr8), *SlSDG5* (chr2), *SlSDG35* (chr12), *SlSDG43* (chr1) located terminally on different chromosomes outside the available markers. We then combined the information about the phenotype of ILs with *HM* expression profiles described in the previous sections.

As a case study, we report the identification of a candidate HM involved in carotenoid biosynthesis in tomato fruits. It should first be noted that the Arabidopsis histone methyltransferase AtSDG8 is required for the expression of the carotenoid isomerase *AtCRTISO*[51]. The tomato homolog of *AtCRTISO* was characterized by Isaacson and colleagues [70] as an essential gene for the production of all *trans*-lycopene. As reported above, our analysis highlighted that two homologs of AtSDG8 occur in tomato, i.e. SlSDG33 and SlSDG34. It should be pointed out that SlSDG33 is a stronger candidate than SlSDG34 as it is involved in *CRTISO-like* regulation and hence in the carotenoid composition of the tomato fruit. Indeed, similar to what is observed for tomato *CRTISO*, *SlSDG33* is upregulated during fruit ripening (see Additional file 13) with a peak of expression in fruit at B and B10. Furthermore, it maps on IL4-3-2 that is reported to have a QTL affecting fruit color, which is known to be dependent on carotenoid biosynthesis [71].

## Conclusions

It is well known that a large genome dataset accelerates gene discovery in plants. In this study, we identified *in silico* 124 HMs in tomato including 32 HATs, 14 HDACs, 52 HMTs, and 26 HDMs. The characterization of HM proteins based on domain annotation was very useful for discovering new family members and new families. Indeed, we revised the canonical family annotation of plant HAGs, reporting the existence in plants of HPA2-like proteins, so far described only in fungi. Moreover, we found that HPA2-like proteins represent the largest group of HAGs both in Arabidopsis and in tomato. Furthermore, we identified a new HAT family, named GLM, revealing that it occurs in 12 plant species. Phylogenetic analysis allowed us to trace the evolutionary history of plant HMs, evidencing their diversification among dicot and monocot species included in this study. By analyzing the expression data of all the *HMs* identified in this study, we were able to provide an overview of the putative role of these genes in tomato development. In this way we supplied useful inputs to discover genes with broader as well as more specific roles. Our datasets might help to address several biological questions and explore the relationship between genomes and phenotypes. Indeed, we propose to combine genome-wide knowledge of *HMs* with the phenotype information of tomato ILs to prioritize candidate genes involved in the process of interest.

## Methods

### Data collection and prediction of tomato HMs

The protein sequences of histone modifiers (HMs) from *Arabidopsis thaliana*, *Oryza sativa* and *Zea mays* were retrieved from ChromDB (http://www.chromdb.org) and are listed in Additional file 14. In order to complete the catalogue of Arabidopsis HAGs and of maize JMJs, the TAIR10 (http://www.arabidopsis.org) and Zmays166 (http://www.phytozome.net/) proteome were downloaded and a BLAST [72] search was then performed using as query the catalytic domain Acetyltransf_1 (PF00583) and different members of Arabidopsis JMJ subfamilies, respectively. The proteins showing only the Acetyltransf_1 and the JMJc catalytic domain (PF02373) were selected. All proteins retrieved were annotated with InterProScan, and for each family of HM a multiple alignment with MUSCLE [73] was performed. The typical domains of each HM family were extracted from the multiple alignments and used to build specific HMM profiles with HMMER v2.1 [74]. The HMM profiles, after calibration, were used as matrix to search for putative HM proteins in the tomato proteome (FASTA file of all predicted proteins of tomato v2.40).

The homologous proteins to SlHAG4 were found by BLAST search against the proteomes of 29 plant organisms (Phytozome v8.0; http://www.phytozome.net). The Interpro database was queried to identify all the proteins showing both the HAT1_N (PF10394) and the MOZ_SAS (PF01853) domains. The list of SlHAG4 homologous proteins and that found in Interpro is reported in Additional file 15.

### HM domain identification

The domain composition of HMs from *Solanum lycopersicon, Arabidopsis thaliana, Oryza sativa* and *Zea mays* was inferred with SMART [75] looking for outlier homologs and PFAM domains.

### Molecular phylogenetic analysis

Phylogeny history of HMs was inferred within Phylogeny.fr environment (http://www.phylogeny.fr/version2_cgi/index.cgi; [76]) Each HM protein group was aligned with MUSCLE [73]. Bayesian phylogeny reconstruction using MrBayes (substitution model = Blosum62; number of generations = 100000; sampling frequency = 100; burnin = 1000) was performed for HAC, HAF, HAM, HDMA, HDT, JMJ and SRT protein families. Maximum Likelihood phylogeny reconstruction was employed for HAG, HDA, PRMT and SDG protein families with PhyML. The bootstrap consensus tree was inferred from 100 replicates [77]. The phylogenetic trees were visualized with FigTree 1.3.1 (http://tree.bio.ed.ac.uk/software/figtree/).

### Expression data visualization

The expression data of tomato *HMs* extracted from dataset of the Tomato Genome Consortium [34] were visualized with Matrix2PNG (http://www.chibi.ubc.ca/matrix2png/bin/matrix2png.cgi; [78]). The expression data of *JMJs, PRMTs* and *SDGs* were normalized to have mean zero and variance one before producing the heat maps. The genes belonging to each HM family were grouped according to their expression profiles in fruits as calculated with MEV 4.8.1 [79,80] by using the Gene Distance Matrix tool.

### Mapping of tomato HM on introgression lines (ILs)

*S. pennellii* IL bins (IL-bins) [81] were visualized on the Sol Genomics Network website (http://solgenomics.net). In order to define the starting and ending point of each IL-bin on the tomato genome, edge molecular marker sequences (MMs) were downloaded. Genome coordinates of MMs were retrieved using BLASTn query against the *Solanum lycopersicon* 2.30 genome (see Additional file 16). The starting coordinate of each tomato *HM* was then compared to coordinates of MMs to establish associations (see Additional file 12).

## Competing interests

The authors declare that they have no competing interests.

## Authors’ contributions

RAC performed the data collection and the analysis of protein domains and ILs, participated in phylogenetic and expression analyses, study design and drafted the manuscript. WS performed the phylogenetics and expression profiles, and participated in bioinformatic analysis design. GC assisted in the interpretation of data. MRE provided advisory support in the bioinformatic analysis and revised the manuscript. CC conceived of the study and revised the manuscript. FMC conceived of and coordinated the study, and revised the manuscript. All authors read and approved the final manuscript.

## Supplementary Material

Additional file 1 **Phylogenetic tree of AT1-domain containing proteins.** Maximum likelihood phylogenetic tree of predicted proteins from *Arabidopsis thaliana* (At) and *Solanum lycopersicon* (Sl) containing an Acetyltrans_1 domain. Bootstrap values higher than 50% are shown. The tree is drawn to scale, with branch lengths measured in the number of substitutions per site. GCN5-like, ELP3-like, HAT1-like and HPA2-like proteins are highlighted in spring green, yellow and green, respectively. Click here for file

Additional file 2 **Phylogenetic tree of GML proteins.** Maximum likelihood phylogenetic tree of predicted proteins from 32 organisms. Bootstrap values are shown for each node. The tree is drawn to scale, with branch lengths measured in the number of substitutions per site. Ac = *Aquilegia coerulea*; Al = *Arabidopsis lyrata*; At = *Arabidopsis thaliana*; Bd = *Brachypodium distachyon*; Br = *Brassica rapa*; Car = *Capsella rubella*; Cc = Citrus clementina; Cis = *Citrus sinensis*; Cp = *Carica papaya*; Cr = *Chlamydomonas rheinhardtii*; Cs= *Cucumis sativus*; Es = *Ectocarpus siliculosus*; Eu = *Eucalyptus grandis*; Gm = *Glycine max*; Lu = *Linum usitatissimum*; Md = *Malus domestica*; Me = *Manihot esculenta*; Mg = *Mimulus guttatus*; Mt = *Medicago truncatula*; Os = *Oryza saliva*; Pp = *Physcomitrella patens*; Pt = *Populus trichocarpa*; Pv = *Phaseolus vulgaris*; Sb = *Sorghum bicolor*; Si = *Setaria italica*; Sl = *Solanum lycopersicon*; Ta = *Trichoplax adhaerens*; Th = *Thellungiella halophila*; Vc = *Volvox cartei*; Vv = *Vitis vinifera*; Zm = *Zea mays*. The proteins showing both the HAT1_N and the MOZ_SAS domain are highlighted in yellow. Click here for file

Additional file 3 **Phylogenetic tree of HAM proteins.** Bayesian phylogenetic tree of HAM predicted proteins from *Arabidopsis thaliana* (At), *Oryza sativa* (Os), *Solanum lycopersicon* (Sl) and *Zea mays* (Zm). Bootstrap values higher than 50% are shown. The tree is drawn to scale, with branch lengths measured in the number of substitutions per site. Click here for file

Additional file 4 **Expression profiles of tomato*HDACs. *** Heat map of RNA-seq expression data from root, leaf, bud, flower, 1cm_fruit, 2cm_fruit, 3cm_fruit, mature green fruit (MG), berry at breaker stage (B) and berry ten days after breaking (B10). *HDAs* with low, middle and high expression values are reported in A, B and C, respectively. The expression values are measured as reads per kilobase of exon model per million mapped reads (RPKM). Click here for file

Additional file 5 **Phylogenetic tree of SRT proteins.** Bayesian phylogenetic tree of SRT predicted proteins from *Arabidopsis thaliana* (At), *Oryza sativa* (Os), *Solanum lycopersicon* (Sl) and *Zea mays* (Zm). Bootstrap values higher than 50% are shown. The tree is drawn to scale, with branch lengths measured in the number of substitutions per site. Click here for file

Additional file 6 **Phylogenetic tree of HDT proteins.** Bayesian phylogenetic tree of HDT predicted proteins from *Arabidopsis thaliana* (At), *Oryza sativa* (Os), *Solanum lycopersicon* (Sl) and *Zea mays* (Zm). Bootstrap values higher than 50% are shown. The tree is drawn to scale, with branch lengths measured in the number of substitutions per site. Click here for file

Additional file 7 **Phylogenetic tree of PRMT proteins.** Maximum likelihood phylogenetic tree of PRMT predicted proteins from *Arabidopsis thaliana* (At), *Oryza sativa* (Os), *Solanum lycopersicon* (Sl) and *Zea mays* (Zm). Bootstrap values higher than 50% are shown. The tree is drawn to scale, with branch lengths measured in the number of substitutions per site. Click here for file

Additional file 8 **Expression profiles of tomato *PRMTs. *** Heat map RNA-seq expression data from root, leaf, bud, flower, 1cm_fruit, 2cm_fruit, 3cm_fruit, mature green fruit (MG), berry at breaker stage (B) and berry ten days after breaking (B10). The expression values are measured as reads per kilobase of exon model per million mapped reads (RPKM). Click here for file

Additional file 9 **Phylogenetic tree of HDMA proteins.** Bayesian phylogenetic tree of HDMA predicted proteins from *Arabidopsis thaliana* (At), *Oryza sativa* (Os), *Solanum lycopersicon* (Sl) and *Zea mays* (Zm). Bootstrap values higher than 50% are shown. The tree is drawn to scale, with branch lengths measured in the number of substitutions per site. Click here for file

Additional file 10 **Expression profiles of tomato ***HMAs. * Heat map of RNA-seq expression data from root, leaf, bud, flower, 1cm_fruit, 2cm_fruit, 3cm_fruit, mature green fruit (MG), berry at breaker stage (B) and berry ten days after breaking (B10). *HDMAs* with low, middle and high expression values are reported in A, B and C, respectively. The expression values are measured as reads per kilobase of exon model per million mapped reads (RPKM). Click here for file

Additional file 11 **Phylogenetic tree of JMJ proteins.** Maximum likelihood phylogenetic tree of JMJ predicted proteins from *Arabidopsis thaliana* (At), *Oryza sativa* (Os), *Solanum lycopersicon* (Sl) and *Zea mays* (Zm). Bootstrap values higher than 50% are shown. The tree is drawn to scale, with branch lengths measured in the number of substitutions per site. Click here for file

Additional file 12 List of the associations between tomato histone modifier genes and introgression lines (ILs). Click here for file

Additional file 13 **Expression profiles of *CRTISO *and putative *SDGs *involved in carotenoid synthesis.** Heat map of *CRTISO, SlSDG33* and *SlSDG34* RNA-seq expression data from 1cm_fruit, 2cm_fruit, 3cm_fruit, mature green fruit (MG), berry at breaker stage (B) and berry ten days after breaking (B10). The expression values are measured as reads per kilobase of exon model per million mapped reads (RPKM). Click here for file

Additional file 14 **List of the histone modifiers from *Arabidopsis thaliana, Oryza sativa *and *Zea mays *used in this study. ** Click here for file

Additional file 15 List of the SlHAG4 homologous sequences used to reconstruct GML evolutionary history. Click here for file

Additional file 16 List of the starting (S) and ending (E) molecular markers, with the corresponding coordinates, used to map tomato ILs. Click here for file

## References

[B1] Fransz P de Jong H From nucleosome to chromosome: a dynamic organization of genetic information Plant J 2011 66 4 17 10.1111/j.1365-313X.2011.04526.x 21443619

[B2] Kouzarides T Chromatin modifications and their function Cell 2007 128 693 705 10.1016/j.cell.2007.02.005 17320507

[B3] Weber M Schübeler D Genomic patterns of DNA methylation: targets and function of an epigenetic mark Curr Opin Cell Biol 2007 19 273 280 10.1016/j.ceb.2007.04.011 17466503

[B4] Sadeh R Allis CD Genome-wide “re”-modeling of nucleosome positions Cell 2011 147 263 266 10.1016/j.cell.2011.09.042 22000006 PMC3207319

[B5] Fuchs J Demidov D Houben A Schubert I Chromosomal histone modification patterns – from conservation to diversity Trends Plant Sci 2006 11 199 208 10.1016/j.tplants.2006.02.008 16546438

[B6] Pandey R Müller A Napoli CA Selinger DA Pikaard CS Richards EJ Bender J Mount DW Jorgensen RA Analysis of histone acetyltransferase and histone deacetylase families of Arabidopsis thaliana suggests functional diversification of chromatin modification among multicellular eukaryotes Nucleic Acids Res 2002 30 5036 5055 10.1093/nar/gkf660 12466527 PMC137973

[B7] Earley KW Shook MS Brower-Toland B Hicks L Pikaard CS In vitro specificities of Arabidopsis co-activator histone acetyltransferases: implications for histone hyperacetylation in gene activation Plant J 2007 52 615 626 10.1111/j.1365-313X.2007.03264.x 17877703

[B8] Utley RT Ikeda K Grant PA Côté J Steger DJ Eberharter A John S Workman JL Transcriptional activators direct histone acetyltransferase complexes to nucleosomes Nature 1998 394 498 502 10.1038/28886 9697775

[B9] Clayton AL Hazzalin CA Mahadevan LC Enhanced histone acetylation and transcription: a dynamic perspective Mol Cell 2006 23 289 296 10.1016/j.molcel.2006.06.017 16885019

[B10] Yang XJ Seto E HATs and HDACs: from structure, function and regulation to novel strategies for therapy and prevention Oncogene 2007 26 5310 5318 10.1038/sj.onc.1210599 17694074

[B11] Hollender C Liu Z Histone deacetylase genes in Arabidopsis development J Integr Plant Biol 2008 50 875 885 10.1111/j.1744-7909.2008.00704.x 18713398

[B12] Tian L Fong MP Wang JJ Wei NE Jiang H Doerge RW Chen ZJ Reversible histone acetylation and deacetylation mediate genome-wide, promoter-dependent and locus-specific changes in gene expression during plant development Genetics 2005 169 337 345 15371352 10.1534/genetics.104.033142 PMC1448893

[B13] Klose RJ Zhang Y Regulation of histone methylation by demethylimination and demethylation Nat Rev Mol Cell Biol 2007 8 307 318 10.1038/nrm2143 17342184

[B14] Jackson JP Lindroth AM Cao X Jacobsen SE Control of CpNpG DNA methylation by the KRYPTONITE histone H3 methyltransferase Nature 2002 416 556 560 10.1038/nature731 11898023

[B15] Jackson JP Johnson L Jasencakova Z Zhang X PerezBurgos L Singh PB Cheng X Schubert I Jenuwein T Jacobsen SE Dimethylation of histone H3 lysine 9 is a critical mark for DNA methylation and gene silencing in Arabidopsis thaliana Chromosoma 2004 112 308 315 10.1007/s00412-004-0275-7 15014946

[B16] Naumann K Fischer A Hofmann I Krauss V Phalke S Irmler K Hause G Aurich AC Dorn R Jenuwein T Reuter G Pivotal role of AtSUVH2 in heterochromatic histone methylation and gene silencing in Arabidopsis EMBO J 2005 24 1418 1429 10.1038/sj.emboj.7600604 15775980 PMC1142535

[B17] Fischer A Hofmann I Naumann K Reuter G Heterochromatin proteins and the control of heterochromatic gene silencing in Arabidopsis J Plant Physiol 2006 163 358 368 10.1016/j.jplph.2005.10.015 16384625

[B18] Ebbs ML Bender J Locus-specific control of DNA methylation by the Arabidopsis SUVH5 histone methyltransferase Plant Cell 2006 18 1166 1176 10.1105/tpc.106.041400 16582009 PMC1456864

[B19] Hennig L Derkacheva M Diversity of Polycomb group complexes in plants: same rules, different players? Trends Genet 2009 25 414 423 10.1016/j.tig.2009.07.002 19716619

[B20] Berr A Shafiq S Shen WH Histone modifications in transcriptional activation during plant development Biochim Biophys Acta 2011 1809 567 576 10.1016/j.bbagrm.2011.07.001 21777708

[B21] Liu C Lu F Cui X Cao X Histone methylation in higher plants Annu Rev Plant Biol 2010 61 395 420 10.1146/annurev.arplant.043008.091939 20192747

[B22] Thorstensen T Grini PE Aalen RB SET domain proteins in plant development Biochim Biophys Acta 2011 1809 407 420 10.1016/j.bbagrm.2011.05.008 21664308

[B23] Kooistra SM Helin K Molecular mechanisms and potential functions of histone demethylases Nat Rev Mol Cell Biol 2012 13 297 311 10.1038/nrg3219 22473470

[B24] Jiang D Yang W He Y Amasino RM Arabidopsis relatives of the human lysine-specific Demethylase1 repress the expression of FWA and FLOWERING LOCUS C and thus promote the floral transition Plant Cell 2007 19 2975 2987 10.1105/tpc.107.052373 17921315 PMC2174716

[B25] Chen X Hu Y Zhou DX Epigenetic gene regulation by plant Jumonji group of histone demethylase Biochim Biophys Acta 2011 1809 421 426 10.1016/j.bbagrm.2011.03.004 21419882

[B26] Lu F Cui X Zhang S Jenuwein T Cao X Arabidopsis REF6 is a histone H3 lysine 27 demethylase Nat Genet 2011 43 715 719 10.1038/ng.854 21642989

[B27] Shi Y Lan F Matson C Mulligan P Whetstine JR Cole PA Casero RA Shi Y Histone demethylation mediated by the nuclear amine oxidase homolog LSD1 Cell 2004 119 941 953 10.1016/j.cell.2004.12.012 15620353

[B28] Jeong JH Song HR Ko JH Jeong YM Kwon YE Seol JH Amasino RM Noh B Noh YS Repression of FLOWERING LOCUS T chromatin by functionally redundant histone H3 lysine 4 demethylases in Arabidopsis PLoS One 2009 4 e8033 10.1371/journal.pone.0008033 19946624 PMC2777508

[B29] Lu F Cui X Zhang S Liu C Cao X JMJ14 is an H3K4 demethylase regulating flowering time in Arabidopsis Cell Res 2010 20 387 390 10.1038/cr.2010.27 20177424

[B30] Chang B Chen Y Zhao Y Bruick RK JMJD6 is a histone arginine demethylase Science 2007 318 444 447 10.1126/science.1145801 17947579

[B31] Pagnussat GC Yu HJ Ngo QA Rajani S Mayalagu S Johnson CS Capron A Xie LF Ye D Sundaresan V Genetic and molecular identification of genes required for female gametophyte development and function in Arabidopsis Development 2005 132 603 614 10.1242/dev.01595 15634699

[B32] Jones MA Harmer S JMJD5 functions in concert with TOC1 in the arabidopsis circadian system Plant Signal Behav 2011 6 445 448 10.4161/psb.6.3.14654 21358285 PMC3142435

[B33] Lu SX Tobin EM Chromatin remodeling and the circadian clock: Jumonji C-domain containing proteins Plant Signal Behav 2011 6 810 814 10.4161/psb.6.6.15171 21617366 PMC3218477

[B34] Tomato Genome Consortium The tomato genome sequence provides insights into fleshy fruit evolution Nature 2012 485 635 641 10.1038/nature11119 22660326 PMC3378239

[B35] Latrasse D Benhamed M Henry Y Domenichini S Kim W Zhou DX Delarue M The MYST histone acetyltransferases are essential for gametophyte development in Arabidopsis BMC Plant Biol 2008 8 12 10.1186/1471-2229-8-12 19040736 PMC2606689

[B36] Kalamaki MS Alexandrou D Lazari D Merkouropoulos G Fotopoulos V Pateraki I Aggelis A Carrillo-López A Rubio-Cabetas MJ Kanellis AK Over-expression of a tomato N-acetyl-L-glutamate synthase gene (SlNAGS1) in Arabidopsis thaliana results in high ornithine levels and increased tolerance in salt and drought stresses J Exp Bot 2009 60 1859 1871 10.1093/jxb/erp072 19357433 PMC2671631

[B37] Perrella G Consiglio MF Aiese-Cigliano R Cremona G Sanchez-Moran E Barra L Errico A Bressan RA Franklin FCH Conicella C Histone hyperacetylation affects meiotic recombination and chromosome segregation in Arabidopsis Plant J 2010 62 796 806 10.1111/j.1365-313X.2010.04191.x 20230492

[B38] Servet C Conde E Silva N Zhou DX Histone acetyltransferase AtGCN5/HAG1 is a versatile regulator of developmental and inducible gene expression in Arabidopsis Mol Plant 2010 3 670 677 10.1093/mp/ssq018 20457643

[B39] Nelissen H Fleury D Bruno L Robles P De Veylder L Traas J Micol JL Van Montagu M Inzé D Van Lijsebettens M The elongata mutants identify a functional Elongator complex in plants with a role in cell proliferation during organ growth Proc Natl Acad Sci USA 2005 102 7754 7759 10.1073/pnas.0502600102 15894610 PMC1140448

[B40] Nelissen H De Groeve S Fleury D Neyt P Bruno L Bitonti MB Vandenbussche F Van der Straeten D Yamaguchi T Tsukaya H Witters E De Jaeger G Houben A Van Lijsebettens M Plant Elongator regulates auxin-related genes during RNA polymerase II transcription elongation Proc Natl Acad Sci USA 2010 107 1678 1683 10.1073/pnas.0913559107 20080602 PMC2824411

[B41] Kojima S Iwasaki M Takahashi H Imai T Matsumura Y Fleury D Van Lijsebettens M Machida Y Machida C Asymmetric leaves2 and Elongator, a histone acetyltransferase complex, mediate the establishment of polarity in leaves of Arabidopsis thaliana Plant Cell Physiol 2011 52 1259 1273 10.1093/pcp/pcr083 21700721

[B42] Han SK Song JD Noh YS Noh B Role of plant CBP/p300-like genes in the regulation of flowering time Plant J 2007 49 103 114 17144897 10.1111/j.1365-313X.2006.02939.x

[B43] Deng W Liu C Pei Y Deng X Niu L Cao X Involvement of the histone acetyltransferase AtHAC1 in the regulation of flowering time via repression of FLOWERING LOCUS C in Arabidopsis Plant Physiol 2007 143 1660 1668 10.1104/pp.107.095521 17416640 PMC1851829

[B44] Alisung MV Yu CW Wu K Phylogenetic analysis, subcellular localization, and expression patterns of RPD3/HDAI family histone deacetylases in plants BMC Plant Biol 2009 9 37 10.1186/1471-2229-9-37 19327164 PMC2671507

[B45] Xu CR Liu C Wang YL Li LC Chen WQ Xu ZH Bai SN Histone acetylation affects expression of cellular patterning genes in the Arabidopsis root epidermis Proc Natl Acad Sci USA 2005 102 14469 14474 10.1073/pnas.0503143102 16176989 PMC1242287

[B46] Tanaka M Kikuchi A Kamada H The Arabidopsis histone deacetylases HDA6 and HDA19 contribute to the repression of embryonic properties after germination Plant Physiol 2008 146 149 161 18024558 10.1104/pp.107.111674 PMC2230551

[B47] Long JA Ohno C Smith ZR Meyerowitz EM TOPLESS regulates apical embryonic fate in Arabidopsis Science 2006 312 1520 1523 10.1126/science.1123841 16763149

[B48] Bond DM Dennis ES Pogson BJ Finnegan EJ Histone acetylation, VERNALIZATION INSENSITIVE 3, FLOWERING LOCUS C, and the vernalization response Mol Plant 2009 2 724 737 10.1093/mp/ssp021 19825652

[B49] Springer NM Napoli CA Selinger DA Pandey R Cone KC Chandler VL Kaeppler HF Kaeppler SM Comparative analysis of SET domain proteins in maize and Arabidopsis reveals multiple duplications preceding the divergence of monocots and dicots Plant Physiol 2003 132 907 925 10.1104/pp.102.013722 12805620 PMC167030

[B50] Sadder M Alsadon A Al-Thamra M Zakri A Al-Doss A Phylogenetic Analysis of SET Domain in Trithorax SlTX1 of Solanum lycopersicum Plant Omics 2011 4 95

[B51] Schmid M Davison TS Henz SR Pape UJ Demar M Vingron M Schölkopf B Weigel D Lohmann JU A gene expression map of Arabidopsis thaliana development Nature Genet 2005 37 501 506 10.1038/ng1543 15806101

[B52] Cazzonelli CI Cuttriss AJ Cossetto SB Pye W Crisp P Whelan J Finnegan EJ Turnbull C Pogson BJ Regulation of carotenoid composition and shoot branching in Arabidopsis by a chromatin modifying histone methyltransferase, SDG8 Plant Cell 2009 21 39 53 10.1105/tpc.108.063131 19174535 PMC2648095

[B53] Cartagena JA Matsunaga S Seki M Kurihara D Yokoyama M Shinozaki K Fujimoto S Azumi Y Uchiyama S Fukui K The Arabidopsis SDG4 contributes to the regulation of pollen tube growth by methylation of histone H3 lysines 4 and 36 in mature pollen Dev Biol 2008 315 355 368 10.1016/j.ydbio.2007.12.016 18252252

[B54] Berr A McCallum EJ Ménard R Meyer D Fuchs J Dong A Shen WH Arabidopsis SET DOMAIN GROUP2 is required for H3K4 trimethylation and is crucial for both sporophyte and gametophyte development Plant Cell 2010 22 3232 3248 10.1105/tpc.110.079962 21037105 PMC2990135

[B55] Guo L Yu Y Law JA Zhang X SET DOMAIN GROUP2 is the major histone H3 lysine 4 trimethyltransferase in Arabidopsis Proc Natl Acad Sci USA 2010 107 18557 18562 10.1073/pnas.1010478107 20937886 PMC2972934

[B56] Ndamukong I Chetram A Saleh A Avramova Z Wall-modifying genes regulated by the Arabidopsis homolog of trithorax, ATX1: repression of the XTH33 gene as a test case Plant J 2009 58 541 553 10.1111/j.1365-313X.2009.03798.x 19154201

[B57] Arrowsmith DA de Silva J Characterisation of two tomato fruit-expressed cDNAs encoding xyloglucan endo-transglycosylase Plant Mol Biol 1995 28 391 403 10.1007/BF00020389 7632911

[B58] Berr A Xu L Gao J Cognat V Steinmetz A Dong A Shen WH SET DOMAIN GROUP25 encodes a histone methyltransferase and is involved in FLOWERING LOCUS C activation and repression of flowering Plant Physiol 2009 151 1476 1485 10.1104/pp.109.143941 19726574 PMC2773071

[B59] Krause CD Yang ZH Kim YS Lee JH Cook JR Pestka S Protein arginine methyltransferases: evolution and assessment of their pharmacological and therapeutic potential Pharmacol Ther 2007 113 50 87 10.1016/j.pharmthera.2006.06.007 17005254

[B60] Yan D Zhang Y Niu L Yuan Y Cao X Identification and characterization of two closely related histone H4 arginine 3 methyltransferases in Arabidopsis thaliana Biochem J 2007 408 113 121 10.1042/BJ20070786 17666011 PMC2049078

[B61] Scebba F De Bastiani M Bernacchia G Andreucci A Galli A Pitto L PRMT11: a new Arabidopsis MBD7 protein partner with arginine methyltransferase activity Plant J 2007 52 210 222 10.1111/j.1365-313X.2007.03238.x 17711414

[B62] Niu L Lu F Pei Y Liu C Cao X Regulation of flowering time by the protein arginine methyltransferase AtPRMT10 EMBO Rep 2007 8 1190 1195 10.1038/sj.embor.7401111 18007657 PMC2267234

[B63] Niu L Zhang Y Pei Y Liu C Cao X Redundant requirement for a pair of PROTEIN ARGININE METHYLTRANSFERASE4 homologs for the proper regulation of Arabidopsis flowering time Plant Physiol 2008 148 490 503 10.1104/pp.108.124727 18660432 PMC2528109

[B64] Pei Y Niu L Lu F Liu C Zhai J Kong X Cao X Mutations in the Type II protein arginine methyltransferase AtPRMT5 result in pleiotropic developmental defects in Arabidopsis Plant Physiol 2007 144 1913 1923 10.1104/pp.107.099531 17573539 PMC1949897

[B65] Zhang Z Zhang S Zhang Y Wang X Li D Li Q Yue M Li Q Zhang YE Xu Y Xue Y Chong K Bao S Arabidopsis floral initiator SKB1 confers high salt tolerance by regulating transcription and pre-mRNA splicing through altering histone H4R3 and small nuclear ribonucleoprotein LSM4 methylation Plant Cell 2011 23 396 411 10.1105/tpc.110.081356 21258002 PMC3051234

[B66] Krichevsky A Gutgarts H Kozlovsky SV Tzfira T Sutton A Sternglanz R Mandel G Citovsky V C2H2 zinc finger-SET histone methyltransferase is a plant-specific chromatin modifier Dev Biol 2007 303 259 269 10.1016/j.ydbio.2006.11.012 17224141 PMC1831845

[B67] Krichevsky A Zaltsman A Kozlovsky SV Tian GW Citovsky V Regulation of Root Elongation by Histone Acetylation in Arabidopsis J Mol Biol 2009 385 45 50 10.1016/j.jmb.2008.09.040 18835563 PMC2650830

[B68] Lu F Li G Cui X Liu C Wang XJ Cao X Comparative analysis of JmjC domain-containing proteins reveals the potential histone demethylases in Arabidopsis and rice J Integr Plant Biol 2008 50 886 896 10.1111/j.1744-7909.2008.00692.x 18713399

[B69] Noh B Lee SH Kim HJ Yi G Shin EA Lee M Jung KJ Doyle MR Amasino RM Noh YS Divergent roles of a pair of homologous jumonji/zinc-finger-class transcription factor proteins in the regulation of Arabidopsis flowering time Plant Cell 2004 16 2601 2613 10.1105/tpc.104.025353 15377760 PMC520958

[B70] Isaacson T Ohad I Beyer P Hirschberg J Analysis in vitro of the enzyme CRTISO establishes a poly-cis-carotenoid biosynthesis pathway in plants Plant Physiol 2004 136 4246 4255 10.1104/pp.104.052092 15557094 PMC535854

[B71] Liu YS Gur A Ronen G Causse M Damidaux R Buret M Hirschberg J Zamir D There is more to tomato fruit colour than candidate carotenoid genes Plant Biotechnol J 2003 1 195 207 10.1046/j.1467-7652.2003.00018.x 17156032

[B72] Altschul SF Gish W Miller W Myers EW Lipman DJ Basic local alignment search tool J Mol Biol 1990 215 403 410 2231712 10.1016/S0022-2836(05)80360-2

[B73] Edgar RC MUSCLE: a multiple sequence alignment method with reduced time and space complexity BMC Bioinformatics 2004 5 113 10.1186/1471-2105-5-113 15318951 PMC517706

[B74] Finn RD Clements J Eddy SR HMMER web server: interactive sequence similarity searching Nucleic Acids Res 2011 39 W29 W37 10.1093/nar/gkr367 21593126 PMC3125773

[B75] Schultz J Milpetz F Bork P Ponting CP SMART, a simple modular architecture research tool: identification of signaling domains Proc Natl Acad Sci USA 1998 95 5857 5864 10.1073/pnas.95.11.5857 9600884 PMC34487

[B76] Dereeper A Guignon V Blanc G Audic S Buffet S Chevenet F Dufayard JF Guindon S Lefort V Lescot M Claverie JM Gascuel O Phylogeny fr: robust phylogenetic analysis for the non-specialist Nucleic Acids Res 2008 36 W465 W469 10.1093/nar/gkn180 18424797 PMC2447785

[B77] Felsenstein J Confidence limits on phylogenies: an approach using the bootstrap Evolution 1985 39 783 791 10.2307/2408678 28561359

[B78] Pavlidis P Noble WS Matrix2png: a utility for visualizing matrix data Bioinformatics 2003 19 295 296 10.1093/bioinformatics/19.2.295 12538257

[B79] Saeed AI Sharov V White J Li J Liang W Bhagabati N Braisted J Klapa M Currier T Thiagarajan M Sturn A Snuffin M Rezantsev A Popov D Ryltsov A Kostukovich E Borisovsky I Liu Z Vinsavich A Trush V Quackenbush J TM4: a free, open-source system for microarray data management and analysis Biotechniques 2003 34 374 378 12613259 10.2144/03342mt01

[B80] Saeed AI Bhagabati NK Braisted JC Liang W Sharov V Howe EA Li J Thiagarajan M White JA Quackenbush J TM4 microarray software suite Methods Enzymol 2006 411 134 193 16939790 10.1016/S0076-6879(06)11009-5

[B81] Eshed Y Zamir D A genomic library of Lycopersicon pennellii in L. esculentum: a tool for fine mapping of genes Euphytica 1994 79 175 179 10.1007/BF00022516

