# Supplementary material for: Genome-wide analysis of histone modifiers in tomato: gaining an insight into their developmental roles
Source: BMC Genomics. 2013 Jan 28;14:57. doi: 10.1186/1471-2164-14-57 (PMC3567966; doi:10.1186/1471-2164-14-57)
Supplement: Additional file 1 — Phylogenetic tree of AT1-domain containing proteins. Maximum likelihood phylogenetic tree of predicted proteins from Arabidopsis thaliana (At) and Solanum lycopersicon (Sl) containing an Acetyltrans_1 domain. Bootstrap values higher than 50% are shown. The tree is drawn to scale, with branch lengths measured in the number of substitutions per site. GCN5-like, ELP3-like, HAT1-like and HPA2-like proteins are highlighted in spring green, yellow and green, respectively. [file 1471-2164-14-57-S1.pdf]

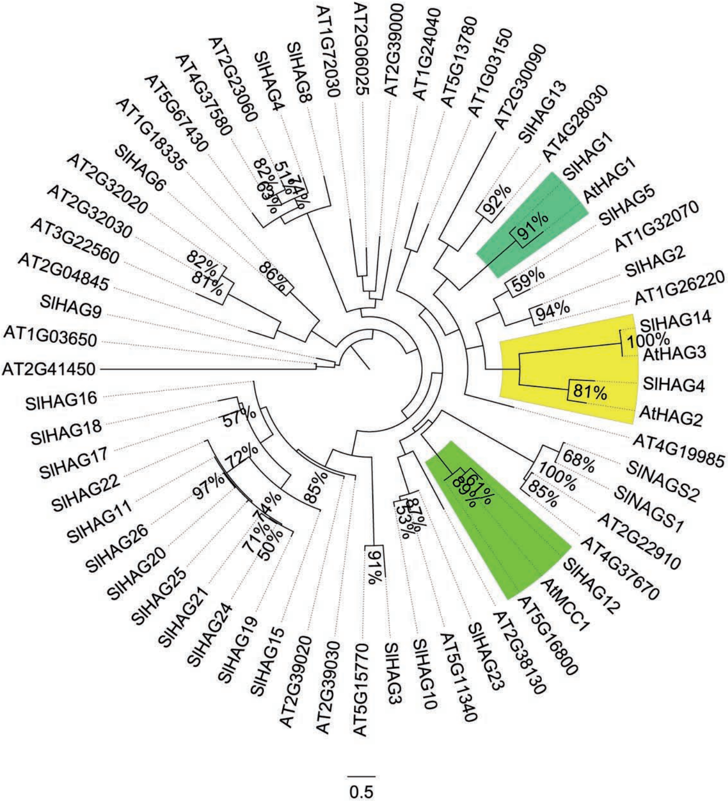

Additional file 1. Phylogenetic tree of AT1-domain containing proteins. Maximum likelihood phylogenetic tree of predicted proteins from *Arabidopsis thaliana* (At) and *Solanum lycopersicon* (Sl) containing an Acetyltrans_1 domain. Bootstrap values higher than 50% are shown. The tree is drawn to scale, with branch lengths measured in the number of substitutions per site. GCN5-like, ELP3-like, HAT1-like and HPA2-like proteins are highlighted in spring green, yellow and green, respectively.
