# Supplementary material for: Genome-wide analysis of histone modifiers in tomato: gaining an insight into their developmental roles
Source: BMC Genomics. 2013 Jan 28;14:57. doi: 10.1186/1471-2164-14-57 (PMC3567966; doi:10.1186/1471-2164-14-57)
Supplement: Additional file 2 — Phylogenetic tree of GML proteins. Maximum likelihood phylogenetic tree of predicted proteins from 32 organisms. Bootstrap values are shown for each node. The tree is drawn to scale, with branch lengths measured in the number of substitutions per site. Ac = Aquilegia coerulea; Al = Arabidopsis lyrata; At = Arabidopsis thaliana; Bd = Brachypodium distachyon; Br = Brassica rapa; Car = Capsella rubella; Cc = Citrus clementina; Cis = Citrus sinensis; Cp = Carica papaya; Cr = Chlamydomonas rheinhardtii; Cs= Cucumis sativus; Es = Ectocarpus siliculosus; Eu = Eucalyptus grandis; Gm = Glycine max; Lu = Linum usitatissimum; Md = Malus domestica; Me = Manihot esculenta; Mg = Mimulus guttatus; Mt = Medicago truncatula; Os = Oryza saliva; Pp = Physcomitrella patens; Pt = Populus trichocarpa; Pv = Phaseolus vulgaris; Sb = Sorghum bicolor; Si = Setaria italica; Sl = Solanum lycopersicon; Ta = Trichoplax adhaerens; Th = Thellungiella halophila; Vc = Volvox cartei; Vv = Vitis vinifera; Zm = Zea mays. The proteins showing both the HAT1_N and the MOZ_SAS domain are highlighted in yellow. [file 1471-2164-14-57-S2.pdf]

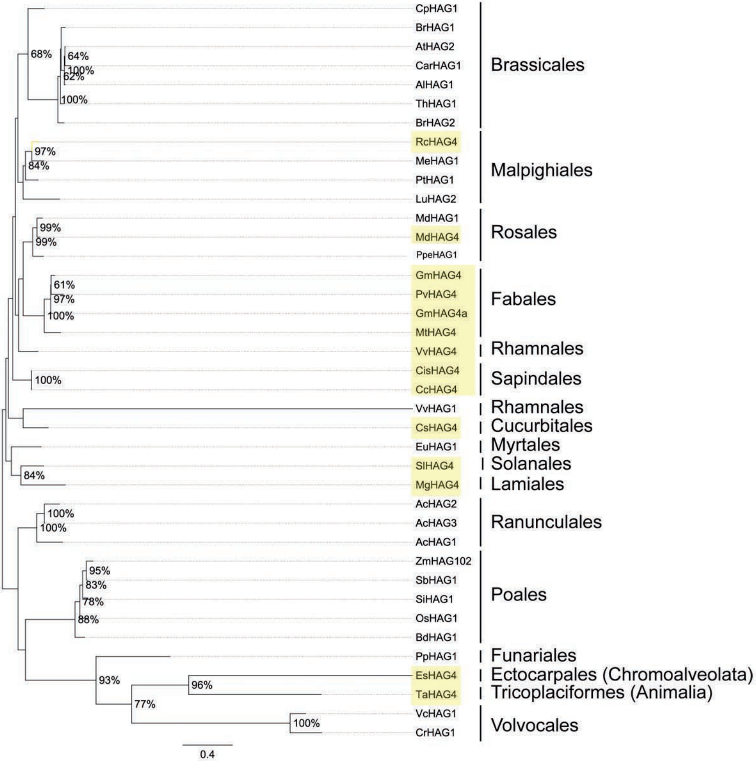

Additional file 2. Phylogenetic tree of GML proteins. Maximum likelihood phylogenetic tree of predicted proteins from 32 organisms. Bootstrap values are shown for each node. The tree is drawn to scale, with branch lengths measured in the number of substitutions per site. Ac = *Aquilegia caerulea*; Al = *Arabidopsis lyrata*; At = *Arabidopsis thaliana*; Bd = *Brachypodium distachyon*; Br = *Brassica rapa*; Car = *Capsella rubella*; Cc = *Citrus clementina*; Cis = *Citrus sinensis*; Cp = *Carica papaya*; Cr = *Chlamydomonas rheinardtii*; Cs= *Cucumis sativus*; Es = *Ectocarpus siliculosus*; Eu = *Eucalyptus grandis*; Gm = *Glycine max*; Lu = *Linum usitatissimum*; Md = *Malus domestica*; Me = *Manihot esculenta*; Mg = *Mimulus guttatus*; Mt = *Medicago truncatula*; Os = *Oryza saliva*; Pp = *Physcomitrella patens*; Pt = *Populus trichocarpa*; Pv = *Phaseolus vulgaris*; Sb = *Sorghum bicolor*; Si = *Setaria italica*; Sl = *Solanum lycopersicon*; Ta = *Trichoplax adhaerens*; Th = *Thellungiella halophila*; Vc = *Volvox cartei*; Vv = *Vitis vinifera*; Zm = *Zea mays*. The proteins showing both the HAT1_N and the MOZ_SAS domain are highlighted in yellow.
