# Supplementary material for: Genome-wide analysis of histone modifiers in tomato: gaining an insight into their developmental roles
Source: BMC Genomics. 2013 Jan 28;14:57. doi: 10.1186/1471-2164-14-57 (PMC3567966; doi:10.1186/1471-2164-14-57)
Supplement: Additional file 5 — Phylogenetic tree of SRT proteins. Bayesian phylogenetic tree of SRT predicted proteins from Arabidopsis thaliana (At), Oryza sativa (Os), Solanum lycopersicon (Sl) and Zea mays (Zm). Bootstrap values higher than 50% are shown. The tree is drawn to scale, with branch lengths measured in the number of substitutions per site. [file 1471-2164-14-57-S5.pdf]

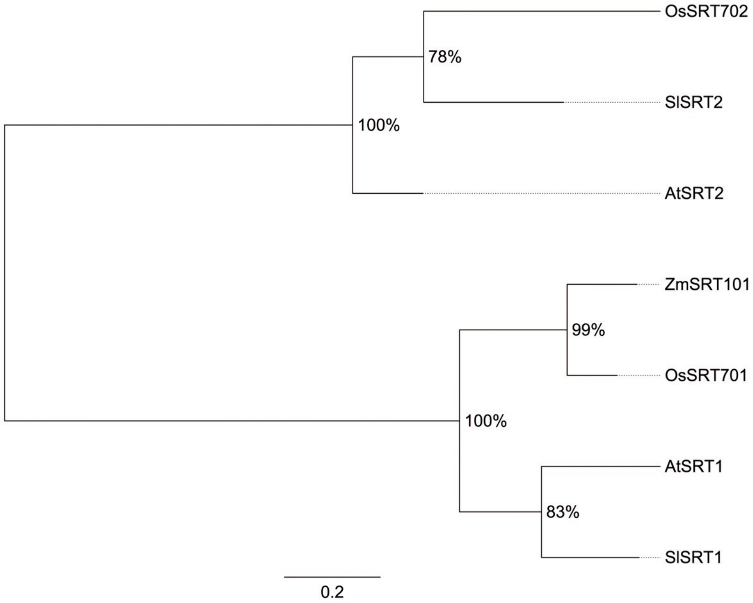

Additional file 5. Phylogenetic tree of SRT proteins. Bayesian phylogenetic tree of SRT predicted proteins from *Arabidopsis thaliana* (At), *Oryza sativa* (Os), *Solanum lycopersicon* (Sl) and *Zea mays* (Zm). Bootstrap values higher than 50% are shown. The tree is drawn to scale, with branch lengths measured in the number of substitutions per site.
