# Supplementary material for: Genome-wide analysis of histone modifiers in tomato: gaining an insight into their developmental roles
Source: BMC Genomics. 2013 Jan 28;14:57. doi: 10.1186/1471-2164-14-57 (PMC3567966; doi:10.1186/1471-2164-14-57)
Supplement: Additional file 8 — Expression profiles of tomato PRMTs. Heat map RNA-seq expression data from root, leaf, bud, flower, 1cm_fruit, 2cm_fruit, 3cm_fruit, mature green fruit (MG), berry at breaker stage (B) and berry ten days after breaking (B10). The expression values are measured as reads per kilobase of exon model per million mapped reads (RPKM). [file 1471-2164-14-57-S8.pdf]

PRMTs

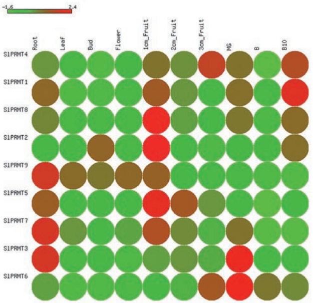

Additional file 8. Expression profiles of tomato *PRMTs*. Heatmap RNA-seq expression data from root, leaf, bud, flower, 1cm_fruit, 2cm_fruit, 3cm_fruit, mature green fruit (MG), berry at breaker stage (B) and berry ten days after breaking (B10). The expression values are measured as reads per kilobase of exon model per million mapped reads (RPKM).
