# Supplementary material for: Genome-wide analysis of histone modifiers in tomato: gaining an insight into their developmental roles
Source: BMC Genomics. 2013 Jan 28;14:57. doi: 10.1186/1471-2164-14-57 (PMC3567966; doi:10.1186/1471-2164-14-57)
Supplement: Additional file 10 — Expression profiles of tomato HMAs. Heat map of RNA-seq expression data from root, leaf, bud, flower, 1cm_fruit, 2cm_fruit, 3cm_fruit, mature green fruit (MG), berry at breaker stage (B) and berry ten days after breaking (B10). HDMAs with low, middle and high expression values are reported in A, B and C, respectively. The expression values are measured as reads per kilobase of exon model per million mapped reads (RPKM). [file 1471-2164-14-57-S10.pdf]

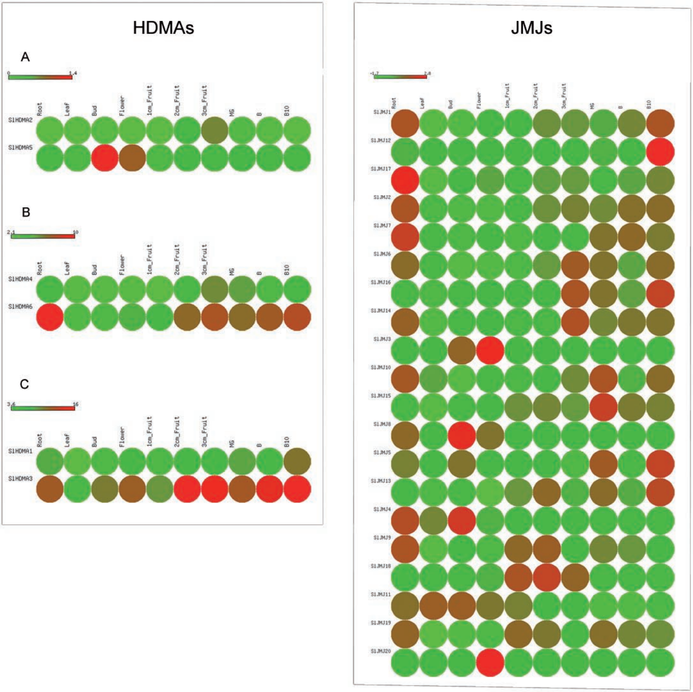

Additional file 10. Expression profiles of tomato *HMAs*. Heatmap of RNA-seq expression data from root, leaf, bud, flower, 1cm_fruit, 2cm_fruit, 3cm_fruit, mature green fruit (MG), berry at breaker stage (B) and berry ten days after breaking (B10). *HDMAs* with low, middle and high expression values are reported in A, B and C, respectively. The expression values are measured as reads per kilobase of exon model per million mapped reads (RPKM).
