# Supplementary material for: Genome-wide analysis of histone modifiers in tomato: gaining an insight into their developmental roles
Source: BMC Genomics. 2013 Jan 28;14:57. doi: 10.1186/1471-2164-14-57 (PMC3567966; doi:10.1186/1471-2164-14-57)
Supplement: Additional file 13 — Expression profiles of CRTISO and putative SDGs involved in carotenoid synthesis. Heat map of CRTISO, SlSDG33 and SlSDG34 RNA-seq expression data from 1cm_fruit, 2cm_fruit, 3cm_fruit, mature green fruit (MG), berry at breaker stage (B) and berry ten days after breaking (B10). The expression values are measured as reads per kilobase of exon model per million mapped reads (RPKM). [file 1471-2164-14-57-S13.pdf]

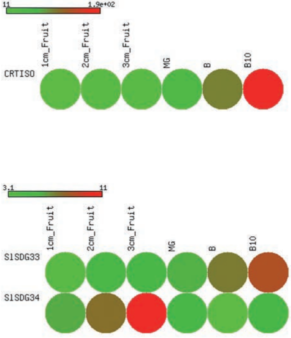

Additional file 13. Expression profiles of *CRTISO* and putative *SDGs* involved in carotenoid synthesis. Heatmap of *CRTISO*, *SlSDG33* and *SlSDG34* RNA-seq expression data from 1cm_fruit, 2cm_fruit, 3cm_fruit, mature green fruit (MG), berry at breaker stage (B) and berry ten days after breaking (B10). The expression values are measured as reads per kilobase of exon model per million mapped reads (RPKM).
